# Specific in situ inflammatory states associate with progression to renal failure in lupus nephritis

**DOI:** 10.1172/JCI155350

**Published:** 2022-07-01

**Authors:** Rebecca Abraham, Madeleine S. Durkee, Junting Ai, Margaret Veselits, Gabriel Casella, Yuta Asano, Anthony Chang, Kichul Ko, Charles Oshinsky, Emily Peninger, Maryellen L. Giger, Marcus R. Clark

**Affiliations:** 1 Section of Rheumatology and Gwen Knapp Center for Lupus and Immunology Research,; 2 Department of Radiology, and; 3 Department of Pathology, University of Chicago, Chicago, Illinois, USA.; 4 Division of Rheumatology, University of Washington, Seattle, Washington, USA.

**Keywords:** Autoimmunity, Adaptive immunity, Lupus

## Abstract

**BACKGROUND:**

In human lupus nephritis (LN), tubulointerstitial inflammation (TII) on biopsy predicts progression to end-stage renal disease (ESRD). However, only about half of patients with moderate-to-severe TII develop ESRD. We hypothesized that this heterogeneity in outcome reflects different underlying inflammatory states. Therefore, we interrogated renal biopsies from LN longitudinal and cross-sectional cohorts.

**METHODS:**

Data were acquired using conventional and highly multiplexed confocal microscopy. To accurately segment cells across whole biopsies, and to understand their spatial relationships, we developed computational pipelines by training and implementing several deep-learning models and other computer vision techniques.

**RESULTS:**

High B cell densities were associated with protection from ESRD. In contrast, high densities of CD8^+^, γδ, and other CD4^–^CD8^–^ T cells were associated with both acute renal failure and progression to ESRD. B cells were often organized into large periglomerular neighborhoods with Tfh cells, while CD4^–^ T cells formed small neighborhoods in the tubulointerstitium, with frequency that predicted progression to ESRD.

**CONCLUSION:**

These data reveal that specific in situ inflammatory states are associated with refractory and progressive renal disease.

**FUNDING:**

This study was funded by the NIH Autoimmunity Centers of Excellence (AI082724), Department of Defense (LRI180083), Alliance for Lupus Research, and NIH awards (S10-OD025081, S10-RR021039, and P30-CA14599).

## Graphical abstract

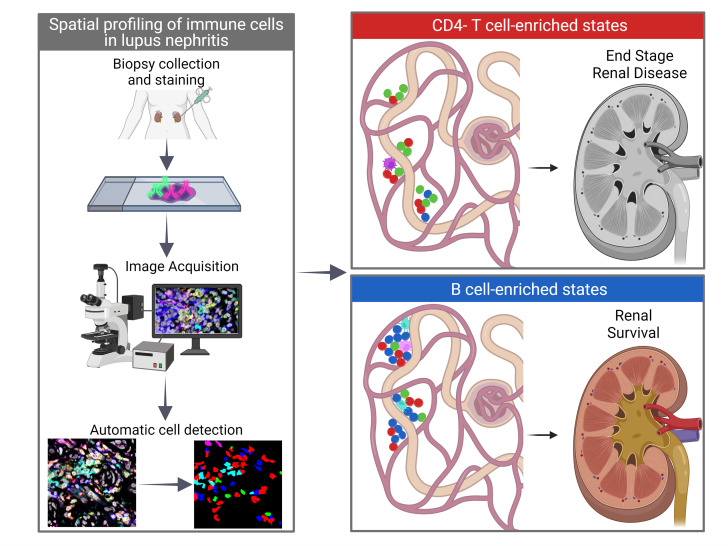

## Introduction

For over 50 years, systemic lupus erythematosus has been thought to result from a break in systemic tolerance and production of pathogenic autoreactive antibodies (1, 2). This canonical model is based on extensive studies of patient blood and spontaneous systemic lupus erythematosus–like animal models (3, 4). In the kidney, the manifestation of systemic autoimmunity is glomerulonephritis (GN). Indeed, lupus nephritis (LN) is usually equated with GN (5). However, tubulointerstitial inflammation (TII) — and not GN — predicts progression to end-stage renal disease (ESRD; refs. 6–9).

Lupus TII is associated with a local immune response very different than the inflammation observed in glomeruli. Indeed, TII is associated with infiltrating B cells, plasma cells, T follicular helper (Tfh) cells, plasmacytoid DCs (pDCs), and myeloid DCs (mDCs), although these cells are rare in LN glomeruli (10–15). These cell subsets are often organized into lymphoid-like architecture in the tubulointerstitium. This phenomenon is associated with local antigen-driven B cell clonal selection (14, 15), suggesting that adaptive immunity in the tubulointerstitium could play a role in driving renal outcomes. There is, therefore, a compelling need to understand in situ adaptive immunity in human LN.

An initial road map to the lupus kidney was provided by the Accelerating Medicines Partnership–funded (AMP-funded) single-cell RNA sequencing (scRNA-Seq) of cells sorted from LN biopsies (16, 17). While these AMP investigations were informative, there were several limitations. The patient sample was small, and the frequency of each immune cell population was not been related to relevant histological features. A larger deficiency of scRNA-Seq is that all spatial information is lost. We do not know how populations spatially relate to each other in the kidney. This lack of spatial information prevents potential functional relationships from being identified.

Previously, in lupus TII, we used conventional immunofluorescence microscopy coupled to evolving computational and machine-learning approaches to characterize the frequency of specific cell populations and identify cell/cell behaviors indicative of cognate immunity (12, 13, 18). However, a systemic analysis of TII and identification of prognostic features has been historically impeded by the complexities of analyzing immunofluorescence data from chronically inflamed kidneys, including tissue autofluorescence due to scarring, antibody cross-reactivity, and patient heterogeneity. Artificial intelligence algorithms have led to significant progress in automated detection and analysis of cells in confocal images (19–22). These approaches have been used successfully in cancer biopsy image analysis (23). However, conventional methods for cell detection and segmentation are not easily generalized to chronically inflamed organs.

Herein, we describe multiple computational pipelines employing deep-learning algorithms that provide high-throughput assessments of cell phenotypes and cellular architectures. These methods have been developed and validated in LN image data sets consisting of both discrete fields of view and entire biopsy sections images. Integration of these data revealed that CD4^–^ T cell populations, comprising CD8^+^, γδ, and double-negative (CD4^–^CD8^–^δ^–^; DN) T cells, often organized into small cellular neighborhoods, are both associated with acute refractory disease and predict progression to renal failure. In contrast, regions of high B cell density were associated with patients who did not progress to renal failure. These and other findings indicate that systemic and in situ autoimmune pathogenic mechanisms are different in LN, and each might require specific targeted therapies.

## Results

### Accurate segmentation of immune cells in LN kidney biopsies.

To probe the relationship between TII and clinical outcome we used a well-characterized cohort of 55 biopsy-proven patients with LN with at least 2 years of follow-up (Supplemental Tables 1 and 2; supplemental material available online with this article; https://doi.org/10.1172/JCI155350DS1). Within this cohort, 19 patients progressed to ESRD (ESRD^+^), requiring either dialysis or transplant within the follow-up period, while 36 did not (ESRD^–^). The ESRD^+^ and ESRD^–^ groups did not differ in length of follow-up, duration of disease, or patient age (Supplemental Figure 1, A–C). Additional information about patient treatment can be found in Supplemental Tables 1 and 2. Thirty-eight patients had moderate or severe TII distributed across both outcome groups. Based on previous studies (6), we hypothesized that differences in renal outcome would be related to differences in in situ adaptive immunity, such as frequency and organization of principal cellular effectors. Therefore, we stained each biopsy for 6 markers, CD3, CD4, CD20, CD11c, BDCA2, and DAPI, to characterize 5 classes of immune cells: CD3^+^CD4^+^ T cells, CD3^+^CD4^–^ T cells, CD20^+^ B cells, BDCA2^+^ pDCs, and CD11c^+^ mDCs. Across the 55 biopsies, we captured all regions of interest (ROIs) with detectable CD3^+^ T cells, resulting in 865 ROIs. Image ROIs were 1024 × 1024 pixels, with a pixel size of 0.1058 μm. These data are referred to as the high-resolution (HR) data set.

**Figure 1:**
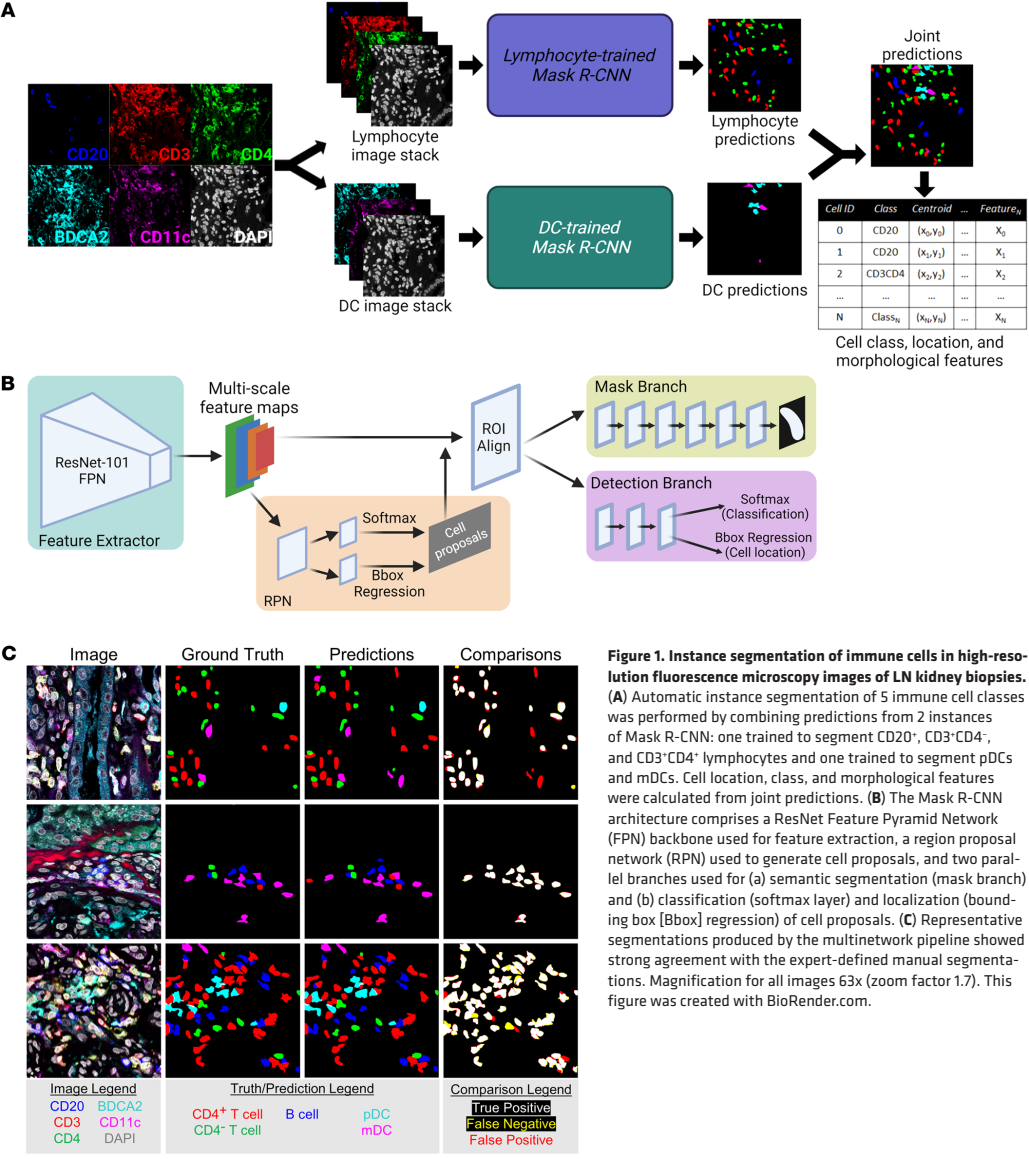
Instance segmentation of immune cells in high-resolution fluorescence microscopy images of LN kidney biopsies. (**A**) Automatic instance segmentation of 5 immune cell classes was performed by combining predictions from 2 instances of Mask R-CNN: one trained to segment CD20^+^, CD3^+^CD4^–^, and CD3^+^CD4^+^ lymphocytes and one trained to segment pDCs and mDCs. Cell location, class, and morphological features were calculated from joint predictions. (**B**) The Mask R-CNN architecture comprises a ResNet Feature Pyramid Network (FPN) backbone used for feature extraction, a region proposal network (RPN) used to generate cell proposals, and two parallel branches used for (a) semantic segmentation (mask branch) and (b) classification (softmax layer) and localization (bounding box [Bbox] regression) of cell proposals. (**C**) Representative segmentations produced by the multinetwork pipeline showed strong agreement with the expert-defined manual segmentations. Magnification for all images 63x (zoom factor 1.7). This figure was created with BioRender.com.

LN is often characterized by chronic and intense inflammation in which accurate cell segmentation can be difficult due to the high cell densities and structured background signal (12, 21). Therefore, we trained deep convolutional neural networks (DCNNs) to perform automatic cell detection, classification, and segmentation (collectively known as instance segmentation) on the HR data set. To achieve optimal performance across all cell classes, we split the 5-class cell detection into two tasks: instance segmentation of lymphocytes and instance segmentation of DCs (Figure 1A). For each task, a separate instance of a region-based DCNN architecture, Mask R-CNN, was independently trained (Figure 1B and ref. 24). Each Mask R-CNN was trained on 246 manually segmented images with a validation set of 65 manually segmented images used for hyperparameter tuning. A cell prediction was defined as a true positive prediction if it had an intersection-over-union (IOU) score of greater than 0.25 with a ground truth cell of the same class. Additionally, all cell predictions with a network confidence score of less than 0.3 were rejected. On a test set of 34 images from patients unique to the training and validation data, the lymphocyte detection network and the DC detection network had F_1_ scores (Equation 3) of 0.75 and 0.62, respectively, while the overall F_1_ score for detection of all 5 cell classes was 0.74, yielding excellent concordance (Figure 1C). The class-specific F_1_ scores for the lymphocyte network, DC network, and the combined predictions are reported in Supplemental Table 3. By implementing DCNNs, we achieved rapid and accurate multiclass instance segmentation.

### Specific in situ immune cell densities associated with progression to renal failure.

**Figure 2:**
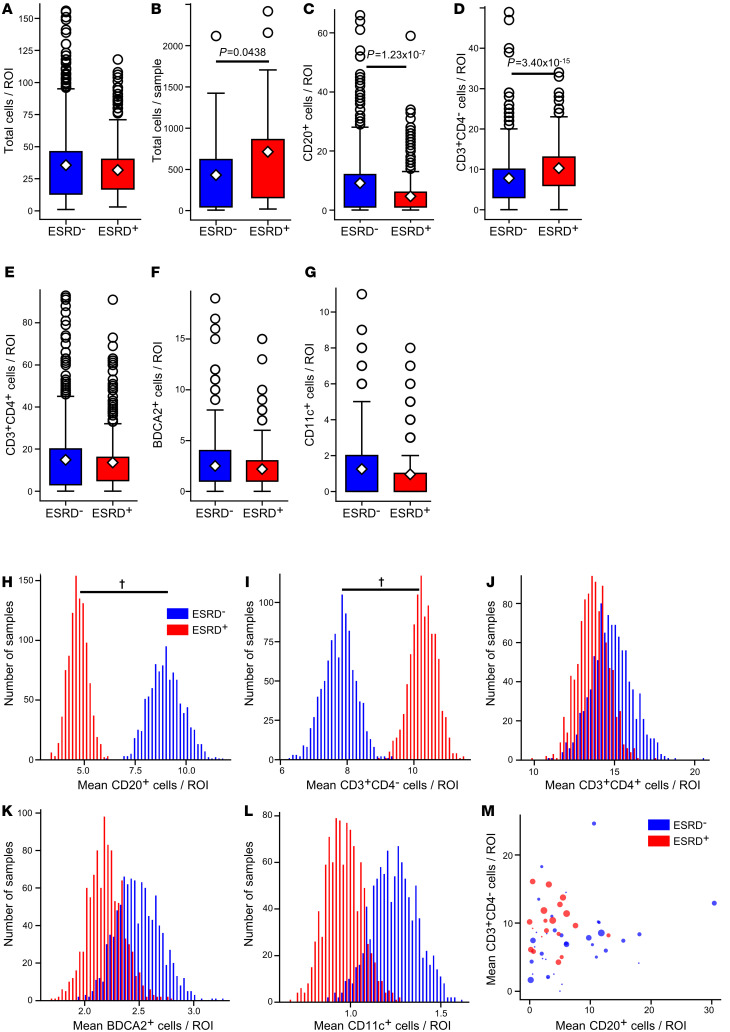
Higher CD4^–^ T cell density and lower B cell density associated with progression to ESRD. (**A**) Local cell density comparison for ESRD^–^ patients (*n =* 437 ROIs) and ESRD^+^ patients (*n =* 428) for all cells. (**B**) Total cells per patient grouped by ESRD status. Local cell density by cell class compared between ESRD^–^ and ESRD^+^ patient for (**C**) CD20^+^ cells, (**D**) CD3^+^CD4^–^ cells, (**E**) CD3^+^CD4^+^ cells, (**F**) BDCA2^+^ cells, and (**G**) CD11c^+^ cells. For all box plots, the population mean is represented by a white diamond, and quartile ranges are defined by the whisker boundaries and upper and lower box boundaries. Outliers are represented as open circles. All cell density comparisons were done with a Mann-Whitney *U* test with a Bonferroni’s correction for multiple comparisons, with significant *P* values noted. Bootstrapped sample means of ESRD^–^ (blue) and ESRD^+^ (red), ROIs for (**H**) CD20^+^ cells/ROI, (**I**) CD3^+^CD4^–^ cells/ROI, (**J**) CD3^+^CD4^+^ cells/ROI, (**K**) BDCA2^+^ cells/ROI, and (**L**) CD11c^+^ cells/ROI. (**M**) Average B cell and CD4^–^ T cell count per ROI for each patient biopsy. Point size is weighted by the TI chronicity score for each patient. **^†^**95% confidence interval does not overlap with 0.

Automatic cell segmentations were used to describe and quantify the spatial distribution of all 5 cell classes in the HR data set. A comparison of overall cell densities (total cells/ROI) in ESRD^–^ and ESRD^+^ patients revealed no significant differences (Figure 2A). However, the total cell count per sample was higher in the ESRD^+^ cohort, reflecting larger overall areas of inflammation (Figure 2B). In contrast to overall cell density, there were differences in the cellular constituents of inflammation between the two patient cohorts. Surprisingly, ROIs from ESRD^–^ patients had higher densities of B cells relative to ROIs from ESRD^+^ patients (Figure 2C). In contrast, ROIs from ESRD^+^ patients had increased densities of CD4^–^ T cells (Figure 2D). There were no significant differences in the densities of CD4^+^ T cells, pDCs, or mDCs between patient cohorts (Figure 2, E–G).

Although there were fewer ESRD^+^ patients, on average these patients had more ROIs captured per biopsy. To mitigate any effect from this class imbalance, we performed a bootstrapping analysis. The pools of ESRD^+^ and ESRD^–^ ROIs were iteratively sampled with replacement 1000 times to produce samples of 200 ROIs from each group (ESRD^+^ and ESRD^–^). The distribution of mean cell densities between ESRD^+^ and ESRD^–^ patients revealed distinct, nonoverlapping peaks for both B cells and CD4^–^ T cells (Figure 2, H and I). In contrast, there was substantial overlap in the distribution of sample means between ESRD^+^ and ESRD^–^ patients for CD4^+^ T cells, pDCs, and mDCs (Figure 2, J–L). The 95% confidence intervals of the difference in means between ESRD^+^ and ESRD^–^ patients revealed for both B cells and CD4^–^ T cells did not cross 0 (Supplemental Figure 2, A and B). In contrast, the 95% confidence interval for the difference in means for the remaining cell types did cross 0 (Supplemental Figure 2, C–E). These data indicate that the observed differences in B cell and CD4^–^ T cell densities between ESRD^+^ and ESRD^–^ patients are robust. Furthermore, our results did not significantly change if the 2 patients who received rituximab were removed (Supplemental Table 1 and data not shown). Therefore, we conclude that high B cell densities are associated with a good prognosis, while high densities of CD4^–^ T cells are associated with progression to renal failure.

When we examine these densities on the patient level, we observed that, in patients with high CD4^–^ T cell densities, B cell densities tended to be low (Figure 2M). As indicated by point size, these tended to be ESRD^+^ patients with higher tubulointerstitial (TI) chronicity scores. The converse appeared true, as patients with higher B cell densities tended to have low TI chronicity scores and be ESRD^–^. These data suggest that lupus TII is associated with two or more distinct inflammatory states, each associated with a different prognosis.

### Patients who present in renal failure have a skewed in situ inflammatory state.

**Figure 3:**
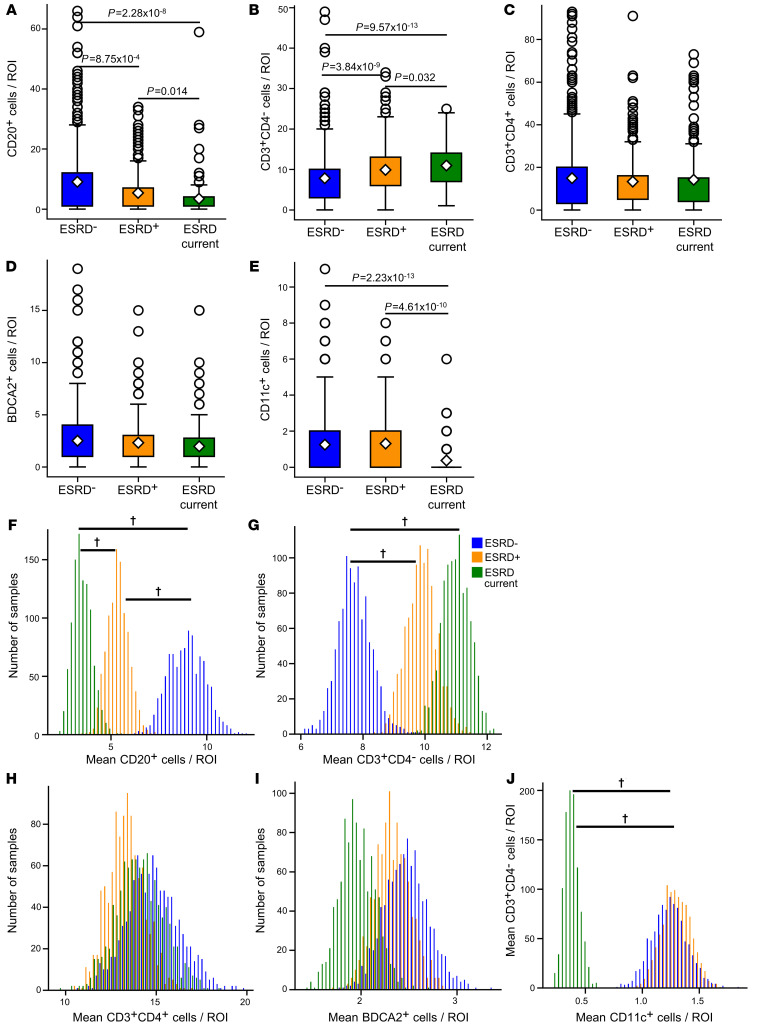
Local cell densities are associated with progressively worse renal outcomes. Local cell density compared across ESRD^–^ patients (*n =* 437 ROIs), ESRD^+^ patients (*n =* 266), and ESRD current patients (*n =* 162) for (**A**) CD20^+^ cells, (**B**) CD3^+^CD4^–^ cells, (**C**) CD3^+^CD4^+^ cells, (**D**) BDCA2^+^ cells, and (**E**) CD11c^+^ cells. For all box plots, the population mean is represented by a white diamond, and quartile ranges are defined by the whisker boundaries and upper and lower box boundaries. Outliers are represented as open circles. All cell density comparisons were done with a Mann-Whitney *U* test with a Bonferroni’s correction for multiple comparisons, with significant *P* values noted. Bootstrapped sample means of ESRD^–^ (blue), ESRD^+^ (orange), and ESRD current (green) ROIs for (**F**) CD20^+^ cells/ROI, (**G**) CD3^+^CD4^–^ cells/ROI, (**H**) CD3^+^CD4^+^ cells/ROI, (**I**) BDCA2^+^ cells/ROI, and (**J**) CD11c^+^ cells/ROI. **^†^**95% confidence interval does not overlap with 0. The data set analyzed in this figure is the same as the data set introduced in Figure 2.

Within the ESRD^+^ group of patients was a small yet distinct cohort of 5 patients that either were in renal failure at the time of biopsy or progressed to renal failure within 2 weeks of biopsy collection. If these patients are treated as their own unique outcome group (ESRD current), differences in the density of specific cell classes become even more apparent (Figure 3). There were progressively fewer B cells/ROI among the ESRD^–^, ESRD^+^, and ESRD current groups, respectively (Figure 3A). The opposite trend was observed for CD4^–^ T cell densities (Figure 3B). In contrast, there were no apparent differences in CD4^+^ T cells or pDCs in the ESRD current patients (Figure 3, C and D). Remarkably, there was a profound depletion of mDCs in the ESRD current cohort (Figure 3E).

A 3-group bootstrapping analysis was performed to assess the effect of class imbalance in patient numbers. ESRD current patients had the lowest mean density of B cells, followed by ESRD^+^ patients, with ESRD^–^ patients having the highest density of B cells (Figure 3F). Confidence intervals for the pairwise differences between bootstrapped samples did not overlap with 0 (Supplemental Figure 3A). An inverse, stepwise relationship was observed for CD4^–^ T cells with progressively higher densities found in the ESRD^+^ and ESRD current patients relative to ESRD^–^ patients (Figure 3G and Supplemental Figure 3B). ESRD current patients were also well separated from the other 2 cohorts with respect to local mDC abundance (Figure 3J and Supplemental Figure 3E). As expected, there were no differences between the 3 groups with respect to CD4^+^ T cells or pDCs (Figure 3, H and I, and Supplemental Figure 3, C and D). These findings indicate that patients with LN that present in renal failure have a skewed inflammatory state with abundant CD4^–^ T cells, relatively few B cells, and a depletion of mDCs.

### Specific cellular neighborhoods associated with progressive and refractory renal disease.

**Figure 4:**
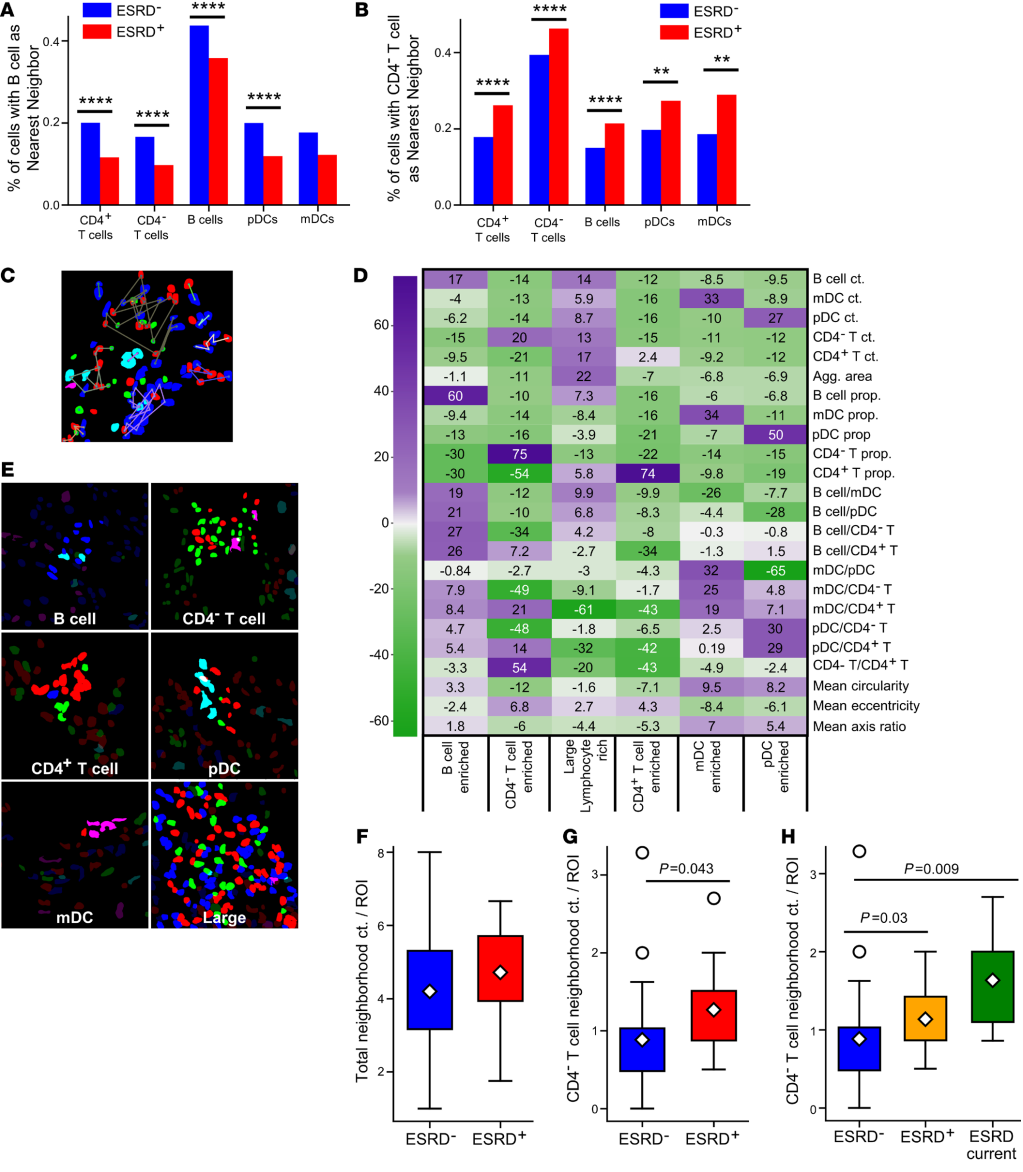
Specific cellular neighborhoods associated with renal failure. Proportions of cells that have (**A**) CD20^+^ B cells and (**B**) CD4^–^ T cells as nearest neighbors in ESRD^+^ and ESRD^–^ patients (χ^2^ test for independence with Bonferroni’s correction for multiple comparisons). (**C**) Neighborhoods of automatically detected cells were detected by DBSCAN. Automatic cell segmentations and representative neighborhoods (highlighted in **E**) are shown for images taken at 63x magnification with a zoom factor of 1.7. (**D**) Heatmap showing test statistics for each feature from leave-one-out *t* tests used to define 6 types of cell neighborhoods, colored by the magnitude of the test statistic. (**E**) Representative neighborhoods from each defined class. (**F** and **G**) The abundance of neighborhoods between the patient cohorts, normalized by the number of ROIs per patient, was compared by Mann-Whitney *U* test with a Bonferroni’s correction for (**F**) all cell neighborhoods and (**G**) CD4^–^ T cell neighborhoods. A 3-group comparison for CD4^–^ neighborhoods, splitting the ESRD^+^ population into ESRD^+^ and ESRD current patients is shown in **H**. Significant *P* values after correcting for multiple comparisons are noted. The data set analyzed in this figure is the same as the data set introduced in Figure 2. For all box plots, the population mean is represented by a white diamond, and quartile ranges are defined by the whisker boundaries and upper and lower box boundaries. Outliers are represented as open circles.

We next explored the relative in situ spatial relationships between the different immune cell classes. First, for every cell in the data set, we identified the nearest neighbor using centroid-to-centroid distances. All cell classes except mDCs were significantly more likely to have a B cell as their nearest neighbor in ESRD^–^ biopsies (Figure 4A). In contrast, all cell classes were significantly more likely to have a CD4^–^ T cell nearest neighbor in ESRD^+^ biopsies (Figure 4B). Additionally, both B cells and CD4^–^ T cells showed a strong propensity for colocalization with cells of the same type.

Local cellular organization was then probed by grouping cells into spatially discrete neighborhoods. DBSCAN, a density-based clustering algorithm (25), was implemented to define cell neighborhoods using a maximum intercellular centroid-to-centroid distance. Variation in this maximum distance between 50 and 150 pixels resulted in a range of neighborhood sizes varying between those that contained just a few cells (50 pixels) to those that encompassed large areas of inflammation (150 pixels) (Figure 4C and Supplemental Figure 4A). A maximum distance of 100 pixels (~10.6 μm) was selected, as this distance approximates a cell body and appeared to capture observable groupings of cells across the data set.

Using this 100-pixel cutoff and a minimum neighborhood size of 2, DBSCAN detected 4022 cell neighborhoods. Each neighborhood was characterized by a set of 24 quantitative features, including cell type frequency, cell type proportion, ratios of cell types, cell shape features, and neighborhood area (Supplemental Figure 4B). K-means clustering was then applied to define classes of neighborhoods, with k = 6 classes determined ideal by bootstrapping cluster descriptors, including the within-cluster sum of squares (WCSS) and the Δ WCSS (Supplemental Figure 4, C and D). The test score from a leave-one-out *t* test approach was used to determine which features or combination of features best distinguished the 6 neighborhood groups (Figure 4D). The most distinctive feature(s) for each group was used to describe the cell neighborhoods as follows: (a) B cell–enriched cluster; (b) CD4^–^ T cell–enriched cluster; (c) large lymphocyte-enriched cluster; (d) CD4^+^ T cell–enriched cluster, (e) mDC-enriched cluster; and (f) pDC-enriched cluster (Figure 4E).

Tertiary lymphoid structures (TLSs) have been previously identified in the context of LN (15). Although we cannot explicitly define TLSs in this data set, we hypothesized that some of the large lymphocyte-enriched neighborhoods might approximate TLSs. For example, we noted that within this group 28.6% of the cells were B cells and 48.3% were CD4^+^ T cells. 96.1% of these neighborhoods met the following criteria: (a) contained at least 20 cells, (b) both B cells and CD4^+^ T cells were represented in the neighborhood, and (c) at least 50% of all cells were B cells and/or CD4^+^ T cells. Therefore, the vast majority of large lymphocyte-enriched neighborhoods have features consistent with TLSs.

We then examined how these 6 classes of neighborhoods were distributed between the ESRD^–^ and ESRD^+^ patients. After normalizing by the number of ROIs captured for each patient, ESRD^–^ and ESRD^+^ patients had no difference in their total neighborhood count per ROI (Figure 4F). However, ESRD^+^ patients had a significantly higher prevalence of CD4^–^ T cell–enriched neighborhoods relative to the ESRD^–^ patients (Figure 4G). The per-ROI prevalence of the other classes of neighborhoods did not correlate with renal outcome (Supplemental Figure 5, A–E). We next examined if the CD4^–^ neighborhoods differed among the ESRD^–^, ESRD^+^, and ESRD current patient groups (Figure 4H). ESRD^+^ and ESRD current patients had a statistically higher prevalence of neighborhoods from the CD4^–^ cluster compared with ESRD^–^ patients. These data demonstrate that, on a per patient basis, the prevalence of small CD4^–^ T cell–enriched neighborhoods is strongly associated with progressive renal disease.

### Cell detection and segmentation in highly multiplexed, full-biopsy images.

To better characterize in situ lymphocyte populations, we performed highly multiplexed (HMP) confocal microscopy on a separate data set of 18 LN biopsies. In this HMP data set, we interrogated a set of 9 markers (CD3, CD4, CD8, ICOS, PD1, FoxP3, CD20, CD138, and DAPI). This HMP panel was obtained using 4-color confocal microscopy and iterative stripping and reprobing (26). Additionally, we imaged full biopsy sections rather than capturing isolated ROIs, thereby facilitating a more complete and unbiased spatial analysis.

**Figure 5:**
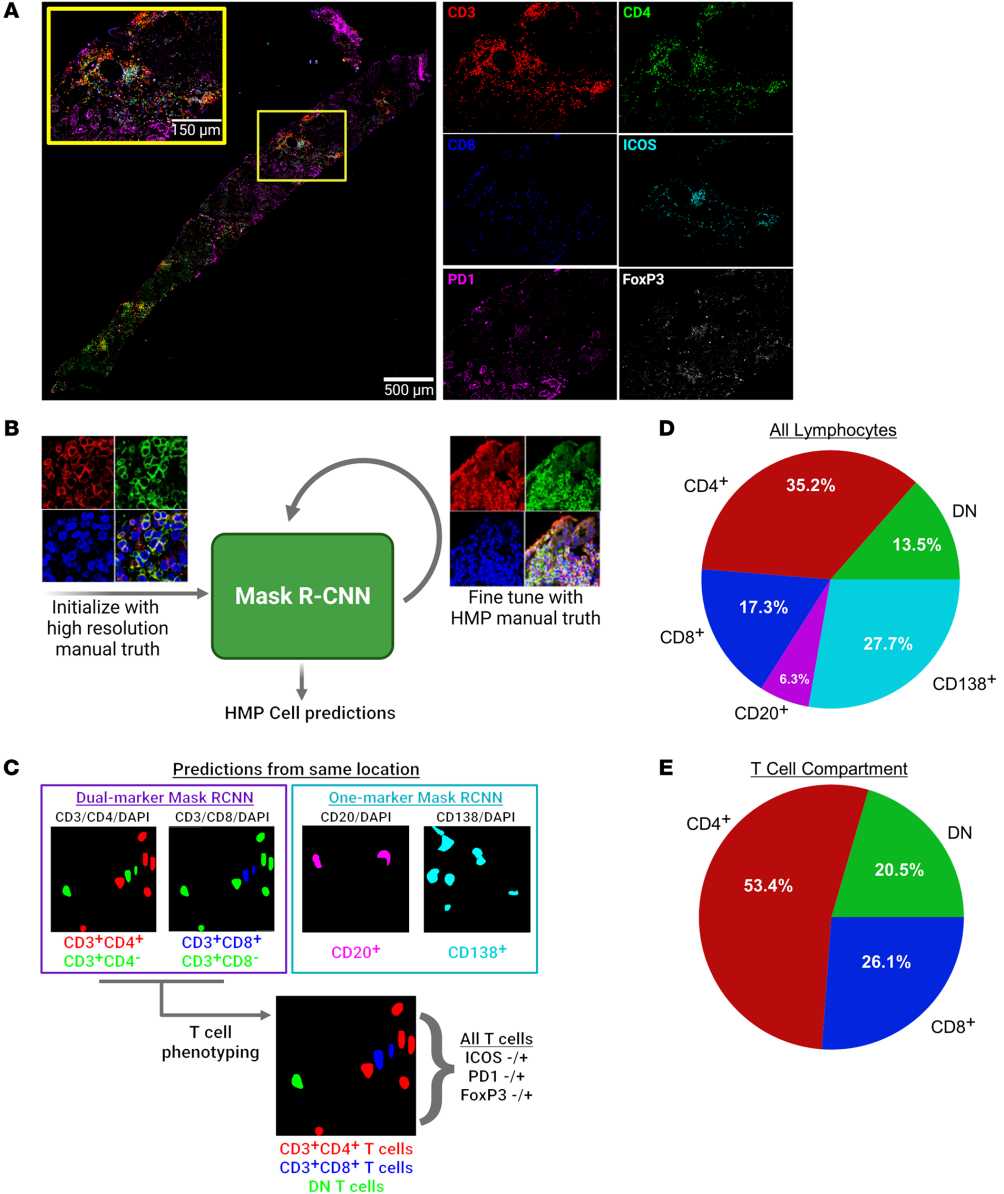
Cell detection, segmentation, and phenotyping in highly multiplexed fluorescence microscopy images. (**A**) Representative composite of a full biopsy section, shown with merged and with isolated panels of CD3, CD4, CD8, ICOS, PD1, and FoxP3. Scale bar: 150 μm; 500 μm (inset). (**B**) Schematic of procedure for training and fine-tuning a Mask R-CNN for instance segmentation of cells in highly multiplexed microscopy images. High resolution, 63x, zoom factor=1.7 (left); multiplexing image: 63x, zoom factor=1 (right). (**C**) Dual-marker and single-marker cell predictions are used to establish base lymphocyte classes. All T cell predictions are further described by ICOS, PD1, and FoxP3 expression. (**D**) Breakdown of frequencies of the 5-base classes in the HMP data set. (**E**) Frequencies of CD4^+^, DN, and CD8^+^ T cells within the T cell compartment. Images in **A**–**C** were created with BioRender.com.

Full biopsy images for all stains were aligned with the DAPI channel (Figure 5A). Two new instances of Mask R-CNN were trained to perform single-marker and dual-marker instance segmentation (Figure 5B). Briefly, ROIs from the HR data set (pixel size = 0.1058 μm) were broken up into 512 × 512 pixel tiles to pretrain each Mask R-CNN. Each network was then fine-tuned using small sets of manually segmented 512 × 512 pixel tiles from the HMP data set (pixel size = 0.221 μm). The single-marker Mask R-CNN was used to predict B cells (CD20^+^) and plasma cells (CD138^+^), while the dual-marker Mask R-CNN was used to predict single-positive and double-positive T cells. The 3 main classes of T cells were determined by combining predictions from a CD3/CD4/DAPI image stack with predictions from a CD3/CD8/DAPI image stack at the same location in the tissue: CD4^+^, CD8^+^, and CD4^–^CD8^–^ (DN) (Figure 5C). The dual-marker Mask R-CNN was also used to generate cell predictions on CD3/ICOS/DAPI and CD3/PD1/DAPI images. The resulting single-positive (CD3^+^ICOS^–^ or CD3^+^PD1^–^) and double-positive (CD3^+^ICOS^+^ or CD3^+^PD1^+^) predictions were used to define ICOS and PD1 expression for every putative T cell in the data set. FoxP3 images were binarized by thresholding individual image tiles. T cell predictions with more than 25% overlap with this binary mask were determined to be FoxP3^+^.

*CD4^–^T cells contain CD8,* γδ*, and other DN T cell populations*. T cells comprised over 65% of predicted lymphocytes in the HMP data set (Figure 5D). Plasma cells were the second-most abundant class, comprising approximately 28% of detected lymphocytes. B cells were least prevalent at only approximately 6%. CD4^+^ T cells were the most abundant cell class across all 5 main classes, making up 35% of detected lymphocytes and over 50% of detected T cells (Figure 5E). Surprisingly, CD8^+^ T cells were only slightly more abundant than DN T cells, comprising approximately 17% of detected lymphocytes and approximately 26% of detected T cells.

**Figure 6:**
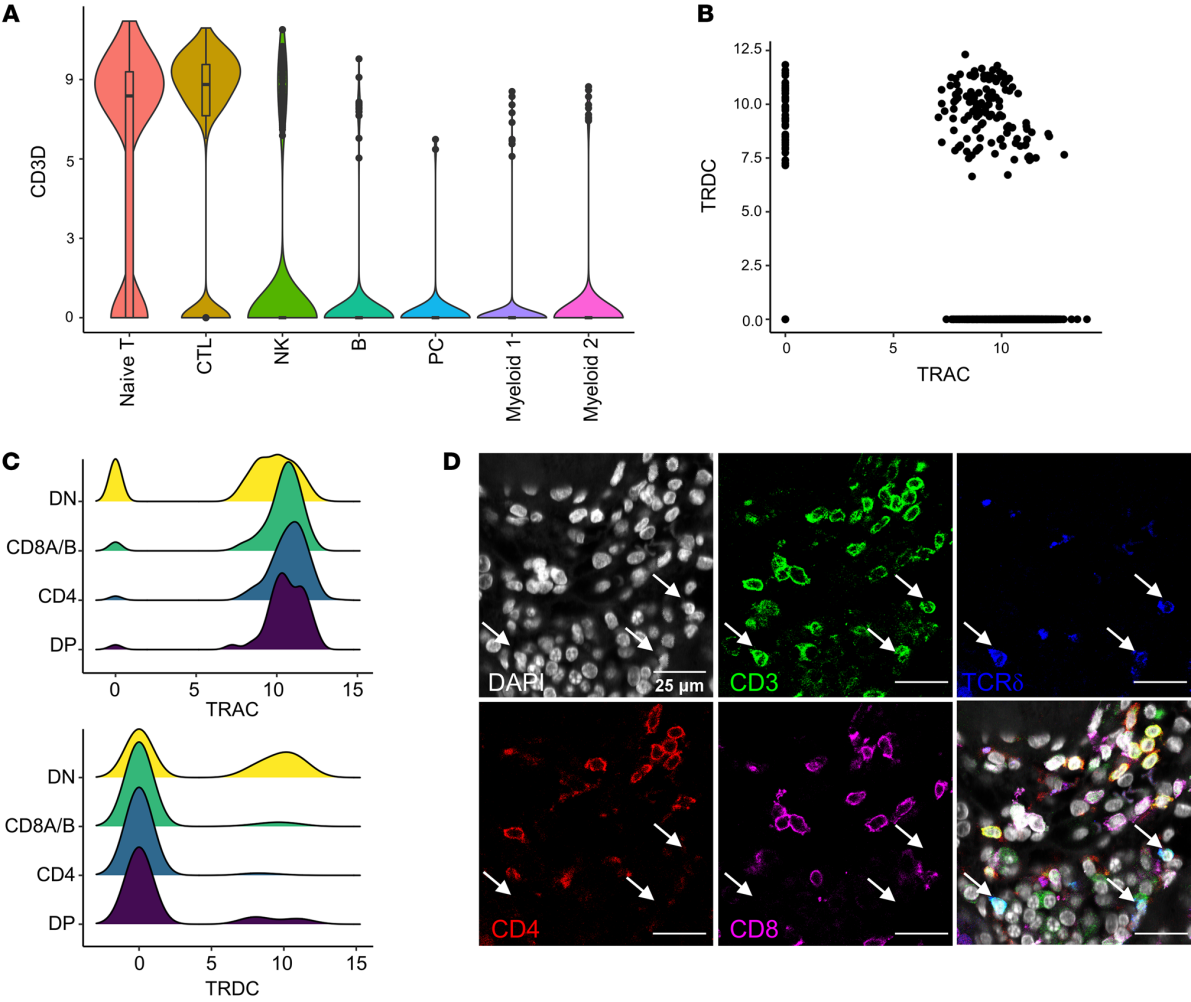
Identifying γδ T cells in LN. (**A**) Distribution of *CD3D* in cell clusters identified in scRNA-Seq data from LN kidney samples. (**B**) Expression of *TRAC* and *TRDC* in T cells identified in scRNA-Seq data. (**C**) Comparison of *TRAC* and *TRDC* expression in identified double-negative (DN), CD8^+^, CD4^+^, and double-positive (DP) T cells. (**D**) Representative image of DN (CD4^–^CD8^–^) γδ (TCRd^+^) T cells in LN biopsy, marked by white arrows. Scale bar: 25 μm.

To further characterize these DN T cells, we interrogated public scRNA-Seq data of immune cells infiltrating the kidneys of patients with LN (16). We identified naive T and CTL clusters in intrarenal immune cells by unsupervised clustering and canonical marker expression (Figure 6A). Within these T cell clusters, 21% were DN, as measured by the unique molecular identifier (UMI) of *CD4*, *CD8A*, and *CD8B* (Supplemental Figure 6A). Several T cell subtypes do not express CD4 nor CD8, including NK T cells and γδ T cells. Indeed, there was a small increase in *CD3D* in cells assigned to the NK cell class, suggesting a NK T cell phenotype. However, there was not a substantial enrichment for NK T cell markers in the DN subset (Supplemental Figure 6B).

Next, we compared TCRα and δ chain expression (*TRAC* and *TRDC*). Some cells were apparently positive for both *TRAC* and *TRDC*, likely due to sequence homology between these genes (Figure 6B). However, *TRAC*^–^ cells and *TRDC*^+^ cells were both enriched in the DN population (Figure 6C). These results suggest that a portion of the DN T cells observed in LN are γδ T cells. To further examine this possibility, we stained 8 LN biopsies with antibodies specific for CD3, CD4, CD8, and TCRδ and imaged 281 ROIs (Figure 6D). Per biopsy, 51.4% ± 21.3% of DN T cells were positive for TCRδ. These findings indicate that a substantial fraction of T cells in LN do not detectably express CD4 or CD8, and approximately half of these DN T cells are γδ T cells.

### In situ distributions of exhausted, regulatory, and helper T cell populations.

We then examined the distributions of ICOS, PD1, and FoxP3 in the T cell subsets. Roughly 30% of CD8^+^ T cells in the HMP data set were PD1^+^ (Supplemental Figure 7A), suggesting an exhausted phenotype. Approximately 25% of CD8^+^ T cells were “exhausted” by the definition of PD1^+^ICOS^–^FoxP3^–^ (27). This is consistent with observations from murine lupus models in which exhausted tissue-infiltrating CD8^+^ T cells are relatively common (28). However, PD1 is only one marker of exhaustion and human lupus renal scRNA-Seq data suggest CD8^+^ T cell exhaustion is infrequent (16).

A surprisingly small percentage (5.41%) of CD4^+^ T cells were FoxP3^+^, while fewer still were also PD1^–^ICOS^–^, suggesting that Tregs comprise only about 2.5% of CD4^+^ T cells (Supplemental Figure 7B). In contrast, even fewer CD8^+^ T cells (1.3%) or DN T cells (0.88%) expressed FoxP3 (Supplemental Figure 7, A and C). Therefore, very few of the tissue-infiltrating CD4^+^ T cells in LN are potentially Tregs.

We additionally identified Tfh cells based on the combination of PD1 and ICOS expression by CD4^+^ T cells (29, 30). 5.05% of the CD4^+^ T cell compartment was PD1^+^ICOS^+^FoxP3^–^. Previous investigations have consistently associated PD1 expression with Tfh-like cells (including T peripheral helper cells) but not necessarily ICOS. Therefore, we applied a less stringent definition of PD1^+^ICOS^+/–^/FoxP3^–^ to identify this cell subset. This Tfh cell phenotype was associated with roughly 30% of the CD4^+^ T cells (Supplemental Figure 7B). Although PD1^+^ICOS^–^FoxP3^–^CD4^+^ T cells could be interpreted as exhausted, we chose to use the more expansive Tfh cell definition in our subsequent analysis.

### Organization of inflammation across whole biopsies.

We next probed potential interacting partners of Tfh cells, Tregs, and exhausted T cells by identifying the class of their nearest neighbors. Most Tregs are closest to other Tregs and other CD4^+^ T cells (Supplemental Figure 7D). In contrast to the expectation that Tfh cells would primarily be in close proximity with CD20^+^ B cells, Tfh cells had other CD4^+^ T cells as their most frequent neighbor, followed by other Tfh cells (Supplemental Figure 7E). Exhausted CD8^+^ T cells were most frequently found near other exhausted CD8^+^ T cells, followed by CD4^+^ T cells and CD8^+^ T cells (Supplemental Figure 7F). Overall, these data demonstrate that across biopsies there is a tendency for the clustering of similar cells together.

**Figure 7:**
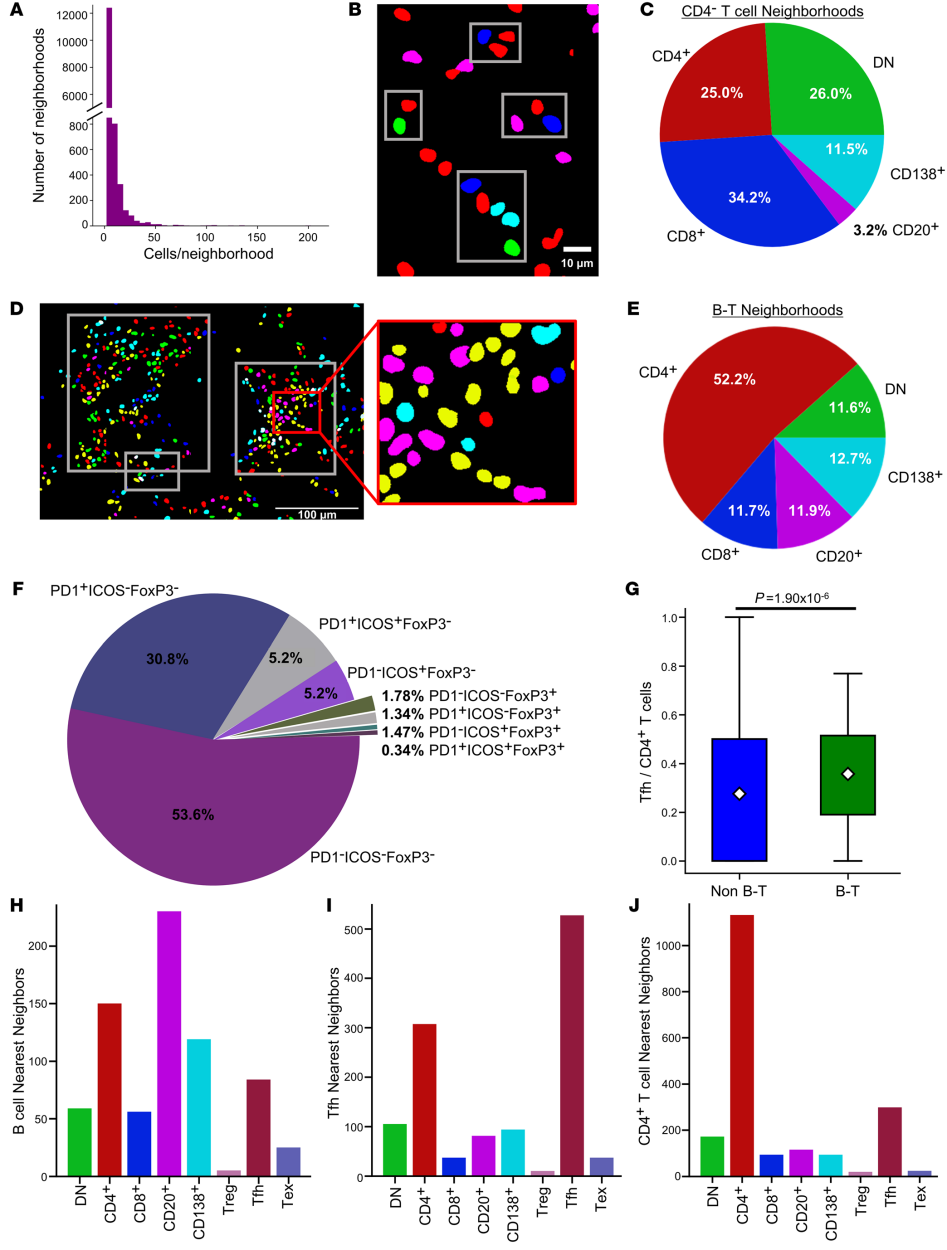
Identification of distinct CD4^–^ and B-T neighborhoods. (**A**) Distribution of sizes of all cell neighborhoods in the HMP data set. (**B**) Representative CD4^–^ clusters (red, CD4^+^ T cells; blue, CD8^+^ T cells; green*,* DN T cells). Scale bar: 10 μm. (**C**) Distribution of the 5 main lymphocyte classes in the CD4^–^ T cell neighborhoods. (**D**) Representative B-T aggregates (outlined by white boxes) (green*,* DN; red, non-Tfh CD4^+^; yellow, Tfh; blue, CD8^+^; magenta, CD20^+^; cyan*,* CD138^+^ cells). Scale bar: 100 μm. (**E**) Distribution of the 5-base classes of lymphocytes in B-T neighborhoods. (**F**) Distribution of CD4^+^ T cell phenotypes in B-T neighborhoods. (**G**) Comparison of proportion of CD4^+^ T cells that are Tfh cells in identified B-T aggregates and non B-T aggregates (Mann-Whitney *U* Test, *P =* 1.9 × 10^-6^). The population mean is represented by a white diamond, and quartile ranges are defined by the whisker boundaries and upper and lower box boundaries. Outliers are represented as open circles. The nearest neighbors of (**H**) CD20^+^ B cells, (**I**) Tfh cells, and (**J**) CD4^+^ T cells within B-T aggregates. The data set analyzed in this figure is the same as the data set introduced in Figure 5.

Cell neighborhoods in the HMP data set were then defined using DBSCAN with a distance cutoff of 50 pixels, roughly 10 μm. Most neighborhoods detected in the HMP data set were small (Figure 7A). However, without the constraint of discrete fields of view, we were able to capture larger neighborhoods, with a maximum neighborhood size of 273 cells, relative to the 147 cell maximum in the HR data set. Given the association of CD4^–^ T cell–enriched neighborhoods with ESRD^+^ patients in the HR data, we investigated similar CD4^–^ neighborhoods in the HMP data. We classified CD4^–^ neighborhoods as those that (a) had less than 20 cells and (b) more than or equal to 25% of their cells were either CD8^+^ or DN T cells, as these criteria captured 99.1% of the CD4^–^ neighborhoods observed in the HR data (Figure 7B). A strong majority of the cells in these neighborhoods were CD4^–^ T cells, including 26% DN T cells and 34.2% CD8^+^ T cells (Figure 7C). There was a weak negative correlation (*R* = –0.35) between the number of DN T cells and CD8^+^ T cells in these neighborhoods, suggesting that DN and CD8^+^ T cells are not proportionally represented in a given neighborhood.

Large B and T cell (B-T) neighborhoods were defined by the set of 3 criteria (as described above) that captured most of the large lymphocyte-rich neighborhoods in the HR data. Of nearly 14,000 neighborhoods in the HMP data set, 111 met these criteria (representative clusters in Figure 7D). Within these B-T neighborhoods, a vast majority of the lymphocytes were T cells, followed by similar proportions of B cells and plasma cells (Figure 7E). Tfh cells made up 36% of CD4^+^ T cells in B-T neighborhoods (Figure 7F), a significant enrichment compared with non–B-T neighborhoods (*P =* 1.9 × 10^-6^, Mann-Whitney) (Figure 7G). As observed across whole biopsies, within these B-T neighborhoods, homotypic proximity predominated. B cells were located near other B cells, followed by plasma cells and CD4^+^ T cells (Figure 7H). Tfh cells in these neighborhoods were most often near other Tfh cells, while overall, Tfh cells were near unspecified CD4^+^ T cells (Figure 7I and Supplemental Figure 7E). Unassigned CD4^+^ T cells in B-T neighborhoods were also most likely to be found near other CD4^+^ T cells, followed by Tfh cells (Figure 7J).

### Cellular neighborhoods are differentially distributed relative to renal structures.

**Figure 8:**
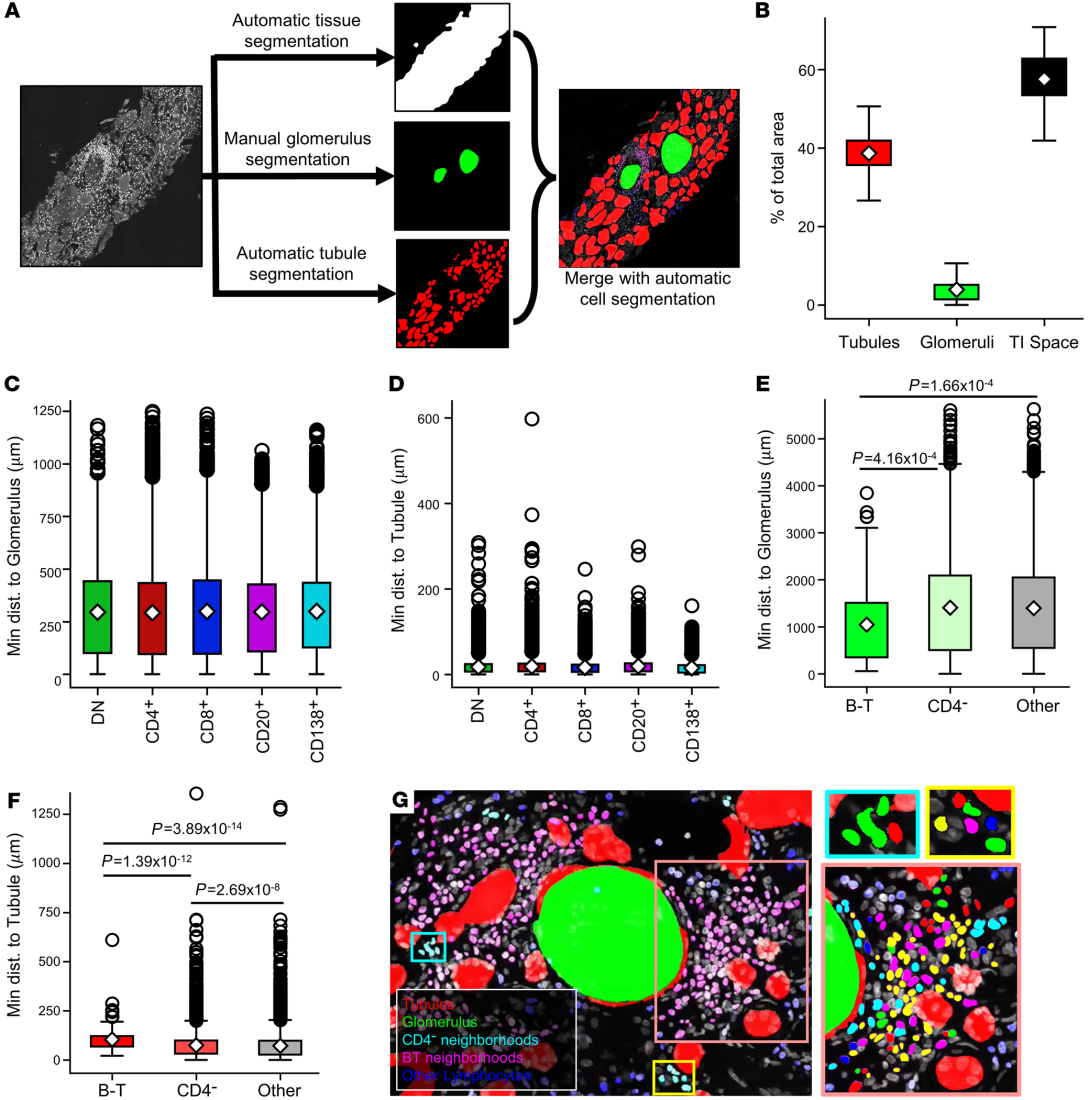
Structural segmentation reveals B-T neighborhood proximity to glomeruli. (**A**) Structural segmentation of biopsies required 3 steps: (a) automatic segmentation of the tissue in the composite was accomplished through filtering and thresholding the DAPI channel, (b) glomeruli were hand-segmented on the DAPI channel of each biopsy, and (c) a U-Net was trained to segment tubular structures (including both tubules and blood vessels) in 512 × 512 DAPI tiles. Structural segmentations were merged with the coordinate space of the detected lymphocytes to calculate proximity to kidney structures (magnification 63x, zoom factor 1 in **A** and **G**). (**B**) The TI space was the largest compartment, followed by tubular structures and then glomeruli. (**C**) Lymphocyte proximity to glomeruli varies slightly across detected classes. (**D**) Minimum distance of detected lymphocytes to a tubule segmentation also varies across class. Means and *P* values for all 2-way comparisons in **C** and **D** are reported in Supplemental Tables 4 and 5. (**E**) B-T neighborhoods were significantly closer to glomeruli than all other neighborhood classifications. (**F**) Conversely, B-T neighborhoods were significantly farther from tubular structures than all other aggregates. CD4^–^ neighborhoods were also significantly farther from tubules than all other non–B-T neighborhoods. The population mean is represented by a white diamond, and quartile ranges are defined by the whisker boundaries and upper and lower box boundaries. Outliers are represented as open circles. (**E** and **F**) Significant *P* values (*P* < 0.05) are noted on plots (Mann-Whitney *U* test with Bonferroni’s correction). (**G**) Representative B-T and CD4^–^ neighborhoods and cell constituents (for zoom panels at right, green*,* DN T cells; red, non-Tfh CD4^+^ T cells; blue, CD8^+^ T cells; magenta, CD20^+^ cells; cyan, CD138^+^ cells; yellow, Tfh cells). The data set analyzed in this figure is the same as the data set introduced in Figure 5.

Whole-biopsy imaging made it possible to characterize the distribution of immune cell populations relative to renal structures. Therefore, we trained a Mask R-CNN to segment the tubular structures in the biopsies, which encompassed proximal and distal tubules, and some vascular structures. Due to a low prevalence, glomeruli were manually segmented (Figure 8A). The relative areas of the tubules, glomeruli, and the resulting TI space were calculated from these structural segmentations (Figure 8B). Glomeruli were indeed much less frequent and comprised the smallest fraction of total tissue. While tubules were abundant, the TI space was the largest structural compartment defined.

The overall proximity of broad lymphocyte classes to tubules and glomeruli was assessed by calculating the minimum distance of a given cell centroid to a pixel defined as either glomerular or tubular. Plasma cells were found to be closer to tubules than all other cell types (Figure 8C and Supplemental Tables 4 and 5). Additionally, B cells and CD4^+^ T cells were significantly farther from tubules than other cell types, although no difference was found between these 2 populations with respect to tubule proximity. Notably, plasma cells were also farther from glomeruli than all other lymphocytes (Figure 8D and Supplemental Tables 4 and 5). CD4^+^ T cells were also closer to glomeruli than CD8^+^ T cells, B cells or plasma cells.

Finally, the locations of the identified CD4^–^ and large B-T neighborhoods relative to the glomeruli and tubules were assessed. On average, the large B-T neighborhoods were significantly closer to glomeruli than both the CD4^–^ neighborhoods and all other uncategorized neighborhoods (Figure 8E). In contrast, B-T neighborhoods were significantly farther from tubules than all other cell neighborhoods. CD4^–^ neighborhoods were closer to tubules than B-T neighborhoods but still significantly farther than the other non–B-T neighborhoods (Figure 8F). These data suggest that there is a preferential aggregation of large B-T neighborhoods near glomeruli, while other quantifiable neighborhoods are dispersed among tubules in the TI space. A representative image of this phenomenon can be seen in Figure 8G. These data suggest that cellular neighborhoods, first identified in discrete fields of view, are spatially organized within the renal cortex.

## Discussion

Canonically, LN is thought of as arising from a systemic break in B cell tolerance that leads to glomerular antibody deposition and inflammation. This model, supported by large bodies of evidence in both humans and mice (10, 31), has led to clinical trials targeting B cells and Tfh cells (32–38). However, these efforts have yielded either incremental or no improvement over the standard of care. By quantifying cellular organization within confocal microscopy images using deep-learning algorithms, we demonstrated that high regional B cell density is associated with a good prognosis. Rather, it is CD4^–^ T cell populations, including CD8^+^, γδ, and other DN T cells, that are associated with refractory disease and progression to renal failure.

The CD4^–^ population was surprisingly heterogeneous. As expected, CD8-expressing cells were common. However, over 40% of the CD4^–^ cells did not express CD8. Of these, approximately 50% expressed the γδ TCR. Reexamination of the AMP data confirmed the presence of intrarenal γδ T cells within the DN T cells. We also observed a substantial population of CD4^–^CD8^–^δ^–^ T cells. These cells appear similar to previously described DN T cells that arise from CD8^+^ self-reactive T cells that have downregulated CD8 expression (39, 40).

It remains to be determined whether a specific CD4^–^ T cell population is associated with progression to renal failure or if these populations share pathogenic roles. Certainly, both CD8^+^ and γδ T cells can be cytolytic and might provide complementary recognition of different classes of autoantigens (41–44). Intrarenal γδ T cells have been implicated in chronic renal disease, though the mechanism is unclear (45, 46). The function of DN T cells is not known, but they might retain cytolytic activity, as they are derived from CD8^+^ T cells. Alternatively, they could be a source of inflammatory cytokines (39). Resolving heterogeneity in these CD4^–^ T cell populations is necessary to identify the populations most closely linked with ESRD.

In addition to yielding information on cell frequencies in tissue, our analytic pipelines provided precise positions of all cells assayed in the biopsy. This allowed us to define cellular neighborhoods and extract quantitative features, including neighborhood size, shape, and cell constituency. Unsupervised clustering revealed that in individual patients small neighborhoods of CD4^–^ T cells were associated with progression to ESRD. These data suggest that understanding immune cell architectures, even in relatively small patient sample cohorts, can identify prognostically important mechanisms.

Our data identified CD4^–^ T cells, including CD8^+^, γδ, and DN T cells, as potentially important therapeutic targets. This association was particularly striking in patients that presented in renal failure. It is possible that the inflammatory phenotype observed in these patients was not a primary state but arose as a secondary consequence of renal damage and scarring. Indeed, the patients who progressed to ESRD generally had higher chronicity scores than the patients who did not. However, 2 of the ESRD current patients (patients 51 and 55) had high densities of CD4^–^ T cells, high activity indices, and relatively low chronicity scores, suggesting that infiltrating CD4^–^ T cells can precede substantial renal damage. These data suggest that patients exhibit distinct, prognostically meaningful, intrarenal inflammatory trajectories.

Unfortunately, in contrast to the B cell/Tfh cell axis, we have limited therapeutic options that specifically target these T cell populations. One of the few classes available is calcineurin inhibitors. The recent success of the calcineurin inhibitor voclosporin in treating some patients with LN is promising (47). We propose that stratifying patients by the constituency and organization of their renal inflammation might identify those most likely to benefit from the addition of T cell–targeting therapies such as voclosporin.

There are several models that might account for the surprising association between dense regions of B cells and good renal outcome. First, B cells might have a previously unappreciated protective role in the context of renal inflammation (48, 49). Alternatively, it is possible that B cells and subsequent local antibody secretion are somewhat benign or neutral compared with other pathological processes in terms of tissue destruction. The final model is that dense B cell regions are responsible for tissue destruction in a subset of patients, but conventional therapies are effective at inhibiting this process. Indeed, most of our patients were treated with high-dose steroids and induced with cytotoxic therapies, most often mycophenolate. These therapies have been demonstrated to deplete B cells and plasma cells (50, 51).

Large neighborhoods of cells were enriched in both B cells and CD4+ T cells, including putative Tfh cells. These neighborhoods have similar features to the T/B aggregates described previously (15). However, our HMP image analysis indicated that these structures are more complex, containing other cell types, including other CD4^+^ T cell and plasma cell populations. These findings cohere with previous studies that suggest an underlying architecture to these large neighborhoods (13). Further work will be needed to understand the rules by which these different neighborhoods organize with respect to each other and the underlying biological processes governing their organization.

Interestingly, pDC prevalence was not associated with renal outcome in our HR data set. As sources of IFN-α, they have been postulated to play a central role in disease pathogenesis (52–55). However, the outcomes of clinical trials of anti–IFN-α antibodies in lupus have been modest (56, 57). In contrast to pDCs, mDCs were depleted in those patients that presented in renal failure. This was an unexpected finding, as CD1c^+^DC-SIGN^+^ DCs and CD141^hi^CLEC9A^+^ DCs in the tubulointerstitium of patients with LN have previously been correlated with fibrosis and poor renal function (58). In addition, periglomerular inflammatory mDCs that drive pathology have been recently described in LN (59), though the mDCs in the tubulointerstitium do not appear to share this phenotype. However, tissue-resident DCs have also been appreciated for their role in enforcing peripheral tolerance (60). Therefore, a subset of DCs might have a role in organ tolerance, even in the context of inflammation.

Furthermore, CD11c is a marker that can capture a range of cells in addition to mDCs, including macrophages and even age-associated or DN B cells (61, 62). In our CNN hierarchy, putative cells expressing both CD20 and CD11c would be classified as B cells; such occurrences were rare. Further work, with multiple additional cell markers, will be needed to resolve the complexity of the CD11c populations.

Using deep-learning and other artificial intelligence algorithms, we achieved robust and accurate cell detection across multiple LN image data sets. This enabled a detailed, accurate spatial analysis of in situ adaptive immunity in LN samples. However, we must acknowledge the limitations of this approach — namely that additional work needs to be done to elucidate the pathological mechanisms at play. We have identified a series of striking associations, but we do not yet have insight into *how* some cells are driving progression to renal failure. For example, it is not feasible to directly test whether the CD4^–^ T cell populations identified in this work are poorly responsive to corticosteroids or mycophenolate therapy. Future work should be focused on validating these findings in a separate cohort of patients from a clinical trial of a B cell– or T cell–specific therapy (e.g., rituximab, voclosporin), which would allow the identified cellular states to be related to therapeutic responses.

Several computer vision methods were implemented to establish an analytical pipeline that addressed experimental, biological, and technical limitations. CNNs trained for instance segmentation detected and classified several immune cell classes with high fidelity not only in sparsely populated images, but also in densely packed images. In previous work, we trained and implemented a single Mask R-CNN for 5-class cell segmentation in LN images (21). By splitting this task into 2 separate networks, we were able to mitigate issues with cell class imbalance and variable stain signatures to improve overall detection performance (63). We also trained and implemented CNNs for rapid and robust image and object filtering to optimize immune cell calling in full biopsy sections. This included a network trained to discriminate between image tiles containing positive cell signal and tissue autofluorescence. We also trained a network to segment tubules in order to reject false-positive plasma cell predictions. By combining these CNNs with thresholding and image registration techniques, we automatically mapped several immune cell classes to full-biopsy sections, enabling a robust spatial analysis of in situ autoimmunity.

We implemented artificial intelligence to quantify immune cell populations in human tissue, thereby extracting rich, nonbiased, spatio-cellular data that allowed identification of unexpected pathogenic mechanisms. Even in a relatively small longitudinal cohort, we were able to resolve patient heterogeneity to identify putative pathogenic processes. Remarkably, specific cell densities provided powerful insights into disease pathogenesis. Understanding how these populations were organized into neighborhoods enabled us to associate cellular features of inflammation with prognosis. Further work needs to be done to identify whether these findings apply to other states of renal inflammation, such as mixed renal rejection or ANCA vasculitis. However, preliminary work indicates that the neural networks and approaches used for LN can be extended to other disease states, such as renal allograft rejection and rheumatoid arthritis. Therefore, our data suggest that using machine-learning-assisted spatial analysis to evaluate the complexity, heterogeneity, and organization of in situ inflammation will help to provide a more quantitative understanding of human autoimmunity. Such knowledge is critical for interpreting and applying the wealth of knowledge we have gained from animal models. It is also likely to identify both new therapeutic targets and those patients in which specific strategies are likely to be beneficial.

## Methods

### Sample staining and image acquisition — HR data set

FFPE kidney biopsies from 55 patients with LN with at least 2 years of clinical follow-up were obtained from the University of Chicago Human Tissue Resource Center (Supplemental Figure 8). FFPE sections were deparaffinized and treated with a citric acid buffer (pH 6.0) for antigen retrieval and blocked with serum prior to antibody staining. Samples were stained with indicated specific antibodies (Supplemental Table 6) and imaged on a Leica SP8 laser scanning confocal microscope at ×63 magnification. Image ROIs were collected in tissue regions with identifiable CD3 signal. Collected images were 1024 × 1024 pixels × 6 channels with a 0.1058 μm pixel size.

### Staining and image acquisition — HMP data set

Samples were stained using a strip and reprobe procedure in which 5 μm thick sections of FFPE biopsy sections were iteratively stained according to a procedure outlined by (ref. 26 and Supplemental Figure 8). Sections were deparaffinized and stained with a combination of primary antibodies and secondary antibodies conjugated with Alexa Fluor 488, 546, and 647 fluorophores (Supplemental Table 6). DAPI was included in every iteration of staining. Each round of staining, samples were imaged using a Caliber ID RS-G4 large-format confocal microscope at a magnification of ×63, resulting in a pixel size of 221 nm. After each round of imaging, samples were stripped as described previously (26) and then reprobed with a new set of primary and secondary antibodies and reimaged until the full marker panel had been imaged.

### Staining and image acquisition — γδ T cells

Eight LN kidney biopsies were stained for CD3, CD4, CD8, TCRδ, and DAPI. Inflamed regions were imaged on the Leica Stellaris 8 confocal microscope, with ×40 magnification and a pixel size of 0.225 μm. 281 ROIs (35 ± 19 per sample) were obtained and then postprocessed with background subtraction, despeckling, and contrast adjustment using ImageJ (NIH). Cells in these images were quantified by manual count.

### Automatic cell detection and segmentation — HR data set

Two instances of a Mask R-CNN architecture (24) were trained to detect and segment cells in this data set. One instance was trained to detect 3 classes of lymphocytes (B cells, CD3^+^CD4^–^ T cells, and CD3^+^CD4^+^ T cells). A second instance was trained to detect 2 classes of DCs (pDCs and mDCs). For this work, ResNet-101 was used as a backbone for the Feature Pyramid Network. Networks were trained with a learning rate of 0.01. Training, validation, and testing data were generated by a single expert. All manually segmented images from a given patient were relegated to the training set (246 images [70%] from 21 unique patients), validation set (62 images [20%] from 4 unique patients), or test set (34 images [10%] from 6 unique patients). Training progress was monitored using Tensorboard, and training was stopped after cell recall stopped improving for all classes. Precision, recall, and F_1_ score were used to evaluate network performance (see equations below). Specifically, these metrics were calculated at the object level, not the pixel level. A predicted object was defined as a true positive if it had an IOU score of greater than 0.25, with a ground truth cell of the same class, and if the network confidence in that predicted object was greater than 0.3. Cell predictions that had an IOU of less than 0.25 with a manual segmentation or had a disagreement in class with an overlapping manual segmentation were defined as false-positive predictions. All manual segmentations that were not matched with a true positive prediction were defined as false negatives. Because instance segmentation is first an object detection task, no true negative objects can be defined. All computation for the HR data set was performed using resources at the University of Chicago Research Computing Center. Each instance of Mask R-CNN was trained on a single GPU compute node containing 4 Nvidia GPUs with 12 GB memory per card, 28 Intel E5-2680v4 CPUs at 2.4 GHz, and 64 GB of system memory. A batch size of 4 images was used for training, distributed across the 4 GPU cards. Networks were trained to the point at which the recall for all cell classes stopped increasing.

 (Equation 1) 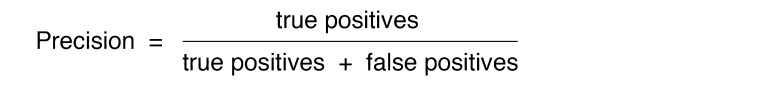


 (Equation 2) 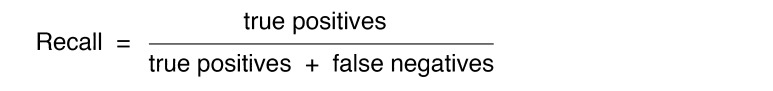


 (Equation 3) 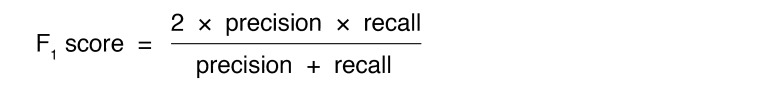


### Automatic cell detection and segmentation — HMP data set

Image strips (1024 × N pixels) from a Caliber ID microscope were stitched together using cross-correlation of image patches at the strip boundaries. These single-channel composites were then aligned with the DAPI channel from the first round of imaging, again through cross-correlation of image patches. Multichannel composites were then broken into 512 × 512 pixel image tiles. All DAPI tiles were passed through a simple image intensity filter to determine if tissue was present at a given location. All tiles at a given location were filtered out of proceeding analyses if the DAPI filter revealed no tissue at that location. This resulted in over unique 18,000 tile locations, with each location containing 9 unique stains.

Instance segmentation of cells was split into two tasks: instance segmentation of T cells (also referred to as dual-marker detection) and instance segmentation of B cells (also referred to as single-marker detection). Instance segmentation performance was evaluated using precision (Equation 1), recall (Equation 2), and F_1_ score (Equation 3).

#### T cell segmentation.

An instance of Mask R-CNN was pretrained to segment single-positive (CD3^+^ CD4^–^) and double-positive (CD3^+^CD4^+^) T cells in 512 × 512 pixel image patches from T cell image stacks (CD3/CD4/DAPI) from the HR data set. This pretrained network was fine-tuned with a small set of 211 T cell image stacks from the HMP data set. The HMP fine-tuning image sets contained CD3/CD4/DAPI, CD3/CD8/DAPI, and CD3/ICOS/DAPI image stacks. This fine-tuning set was split into training (169 images, 80%), validation (21 images, 10%), and testing (21 images, 10%) sets, with all images from a given patient confined to a specific set. For fine-tuning, weights were permitted to adjust for all convolutional, max-pooling, and fully connected layers of the pretrained Mask R-CNN. The fine-tuned T cell network was used to make predictions for CD3/CD4/DAPI, CD3/CD8/DAPI, CD3/ICOS/DAPI, and CD3/PD1/DAPI images. The trained dual-marker network had an average F_1_ score of 0.85 on all single-positive cell predictions (i.e., CD3^+^CD4^–^ and CD3^+^CD8^–^) and an average F_1_ score of 0.92 on all double-positive cell predictions (i.e., CD3^+^CD4^+^ and CD3^+^CD8^+^).

#### B cell segmentation.

An instance of Mask R-CNN was pretrained to segment B cells in 512 × 512 pixel image patches generated from B cell image stacks (CD20/DAPI) from the HR data set. This pretrained network was fine-tuned with a set of 79 B cell image stacks from the HMP data set. This fine-tuning set was split into training (63 images, 80%), validation (8 images, 10%), and testing (8 images, 10%) sets, with all images from a given patient confined to a specific set. For fine-tuning, the weights were permitted to be adjusted for all convolutional, max-pooling, and fully connected layers of the pretrained Mask R-CNN. The fine-tuned B cell network was used to make predictions for B cell (CD20) and plasma cell images (CD138). The trained single-marker network had and average F_1_ score of 0.87.

### Tubule segmentation

An instance of Mask R-CNN was trained to segment tubular structures, including blood vessels and tubules, in the HMP data set. 300 DAPI tiles (512 × 512 pixels) from 18 patients were manually annotated by a single expert. Manually segmented images were separated into training, validation, and testing sets as follows: 240 images in the training set (80%), 30 images in the validation set (10%), and 30 images in the test set (10%). Data augmentation consisted of random horizontal and vertical flips and rotations. Performance of the tubule segmentation network was assessed at the pixel level, with the trained network yielding an average recall (Equation 2) of 0.74 and an average precision (Equation 1) of 0.79 on the test set of tubule images.

All computation associated with the HMP data set was performed on the MEL computational server in the Radiomics and Machine Learning Facility at the University of Chicago. MEL contains 256 Xeon Gold 6130 CPU cores, 3 TB of DDR4 ECC RAM memory, 24 TB of NVMe SSD storage, and 16 Nvidia Tesla V100 32GB GPU accelerators.

### Defining cell neighborhoods through density-based clustering

Cells in both data sets were assigned to clusters using the sklearn (version 0.23.2) implementation of Density-Based Spatial Clustering of Applications with Noise (DBSCAN; ref. 25), using an epsilon of roughly 10 μm, corresponding to 100 pixels in the HR data set and 50 pixels in the HMP data set and a minimum cluster size of 2. In the HR data set, 24 features of cellular constituency and cell/neighborhood shape were extracted for each cluster, and K-means clustering was then applied to define classes of neighborhoods. The neighborhoods were split into 6 classes, as determined ideal by bootstrapping cluster descriptors, including the WCSS and the Δ WCSS. The types were characterized using a leave-one-out *t* test to identify which features of each type of neighborhood distinguished it from the other neighborhoods. In this procedure, the current cluster of reference was treated as the alternative group, all remaining clusters were then binned together as the reference group, and then a *t* test for was applied for all features used to describe the neighborhoods.

### Structural segmentation of kidney biopsies

Full-biopsy segmentation was performed on the DAPI channel for all kidney biopsies. The fully stitched DAPI composite was γ-corrected to increase brightness and then filtered with a Gaussian kernel. The filtered image was normalized and thresholded to create a binary mask of all tissue in the composite. Glomerular segmentation was performed by hand. Due to the relatively low prevalence of glomeruli in this data set, there was not enough glomeruli to train an algorithm to define glomeruli. Tubular structures, including both tubules and blood vessels, were segmented on the DAPI channel of all samples using the Mask R-CNN trained for tubule segmentation (as described above). No markers were included in the staining panel to explicitly identify tubules and blood vessels, and the signatures of these 2 structures are very similar in the DAPI channel. Therefore, the “tubule” network is trained to segment general tubular structures, which include proximal tubules, distal tubules, and blood vessels.

### Spatial analyses

All other spatial analyses were performed using Python (3.7.9) and the following packages: pandas (1.2.2) (https://pandas.pydata.org/), numpy (1.19.2) (https://numpy.org/), sklearn (0.23.2) (https://scikit-learn.org/stable/), scipy (1.6.1) (https://scipy.org/), and tifffile (2021.1.14) (https://pypi.org/project/tifffile/2021.1.14/). Plotting was performed with matplotlib (3.3.2) (https://matplotlib.org/) and seaborn (0.11.1) (https://seaborn.pydata.org/). The nearest-neighbors calculation was performed by iterating through every cell in the data set and identifying the class of the closest cell by centroid-to-centroid distance.

In the HMP data set, coordinates of the cells in the tiles were adjusted to a composite-level coordinate system by shifting the tile-level coordinates based on the location of the tile in the composite. All subsequent calculations around the distribution of cells in tissue were based on these composite-level locations.

### RNA-Seq analysis

scRNA-Seq data for human LN tissue were obtained from the ImmPort repository (accession code SDY997, “SDY997_EXP15176_celseq_matrix_ ru10_molecules.tsv” raw data file). Quality control was performed according to Arazi et al. (16), such that cells were removed from the analysis if they expressed fewer than 1000 or more than 5000 genes or if more than 25% of the total UMIs mapped to mitochondrial genes. Gene expression values were normalized to library size (UMI count per million) and scaled by log_2_. Clustering implemented in Seurat 3.2.2 and canonical marker expression were used to identify cellular subsets. T cells were analyzed if they were assigned to the “naive T” or “CTL” clusters. T cells were categorized based on *CD4*, *CD8A*, and *CD8B* expression. Cells were categorized as “CD4” when they had detectable expression of *CD4* transcripts but no *CD8A* or *CD8B*. They were instead categorized as “CD8” when they had detectable *CD8A* and/or *CD8B* expression with no detectable *CD4* transcripts. Cells were categorized as double-positive or DN when they had both/neither *CD4* and/nor *CD8A/B*. t-SNE was performed by Rtsne (0.15). Plots were generated by ggplot2 (3.3.2) and ggridges (0.5.2).

### Data and materials availability

Code used for the cellular segmentation and spatial analysis can be found at the following repository: https://github.com/durkeems13/LN_image_analysis (commit id: 9281d32).

### Statistics

Differences between the groups of patients or groups of cellular neighborhoods were evaluated using the Mann-Whitney *U* test with the Bonferroni’s correction for multiple comparisons. Differences in proportions were evaluated using the χ^2^ test for independence with Bonferroni’s correction for multiple comparisons. Data are shown as the mean ± SEM.

The bootstrapping analysis to validate the differences between the groups of patients in the HR data set was performed by sampling the individual pools of ROIs with replacement to produce samples of 200 ROIs for each of the patient cohorts in the 2-group analysis (ESRD^+^ vs. ESRD^–^) and 150 ROIs for each of the patient cohorts in the 3-group analysis (ESRD^+^ vs. ESRD^–^ vs. ESRD current). For each pair of groups, the mean for each random sample was calculated as well as the difference between the 2 group means. This procedure was repeated 1000 times to produce distributions of the difference in means between the groups. The 2 populations were considered to be significantly different from each other if the 95% confidence interval of the differences in means that did not overlap with 0.

### Study approval

This study, which used only deidentified human samples, was approved by the University of Chicago Institutional Review Board, with protocol number 15065.

## Author contributions

RA, MSD, and MRC conceptualized the study. RA, MSD, JA, MV, GC, YA, MLG, and MRC developed the methodology. RA, MSD, GC, and YA developed software. RA, MSD, AC, KK, EP, and CO provided clinical data. MRC and MLG acquired funding. MRC and MLG supervised the study. RA, MSD, JA, MV, GC, YA, and MRC wrote the original draft of the manuscript, and RA, MSD, JA, MV, GC, YA, MLG, and MRC reviewed, and edited the manuscript. First authorship was split between RA and MSD, with RA named first, because MSD led the development and implementation of the computer vision techniques, and RA led the spatial analyses of cells that comprise the bulk of the biological findings in the results section.

## Supplementary Material

Supplemental data

Trial reporting checklists

ICMJE disclosure forms

Supplemental table 1

## Version 1 05/24/2022

In-Press Preview

## Version 2 07/01/2022

Electronic publication
