# Supplementary material for: Specific in situ inflammatory states associate with progression to renal failure in lupus nephritis
Source: J Clin Invest. 2022 Jul 1;132(13):e155350. doi: 10.1172/JCI155350 (PMC9246394; doi:10.1172/JCI155350)
Supplement: Supplemental data [file jci-132-155350-s174.pdf]

Supplementary Figures and Tables

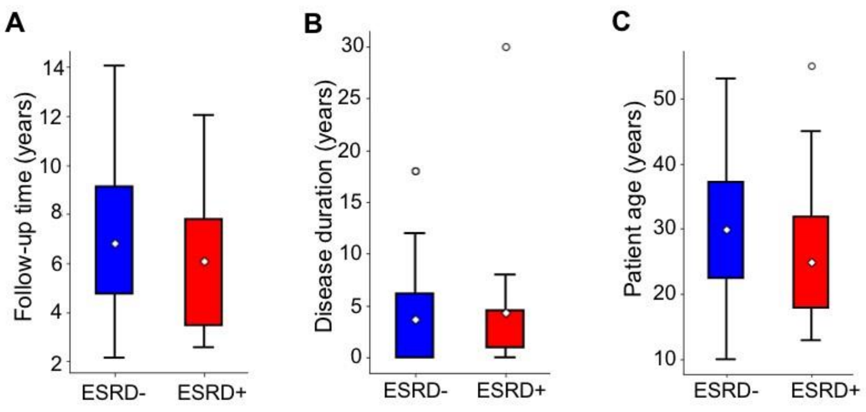

Supplementary Figure 1. Clinical characteristics of patient cohort. ESRD+ and ESRD- patients did not differ in A) Duration of follow up period (p=0.678), B) Disease duration (p=0.819), or C) Patient age (p=0.096). (Mann-Whitney U Test with Bonferroni correction for multiple comparisons)

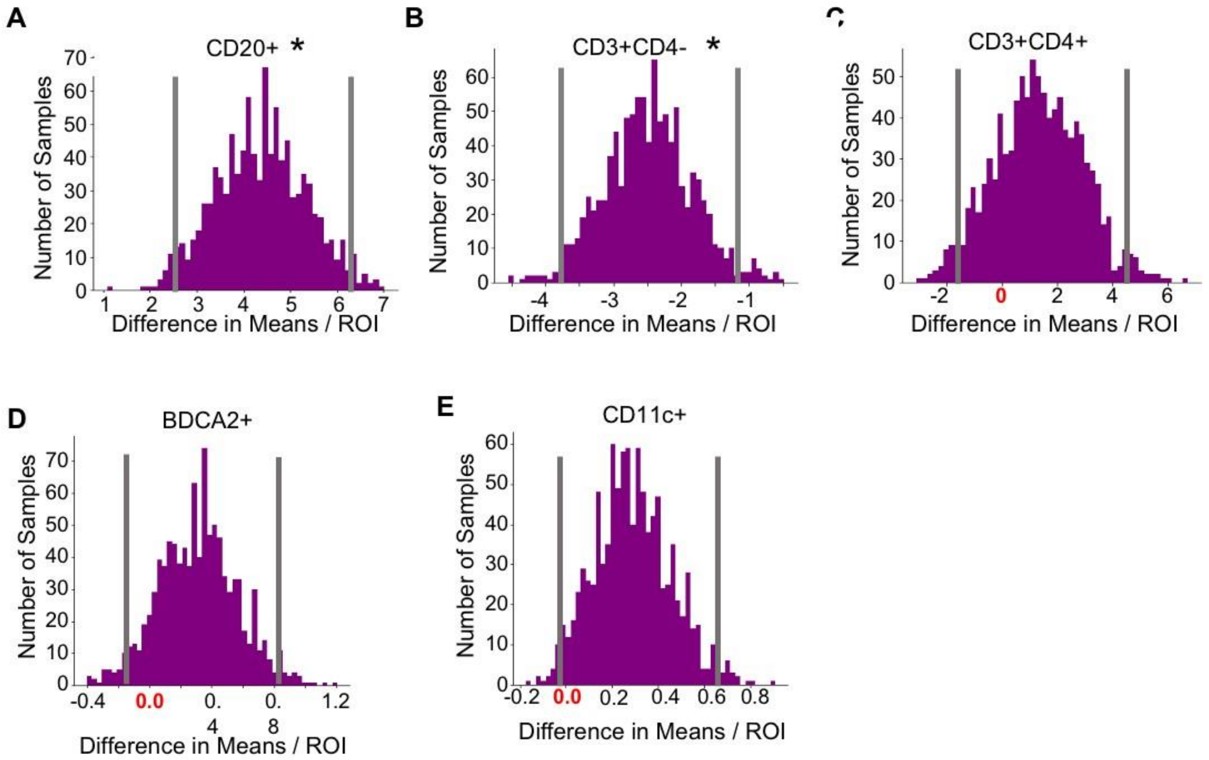

Supplementary Figure 2. **Distribution of the difference in means for indicated cells/ROI comparing ESRD+ vs ESRD-.** Difference in means for all iterations of bootstrapping are shown for A) CD20+, B) CD3+CD4-, C) CD3+CD4+. D) CD11c+, E) BDCA2+ cells. Vertical grey lines denote 95% confidence intervals, stars denote the confidence intervals that do not overlap with zero.

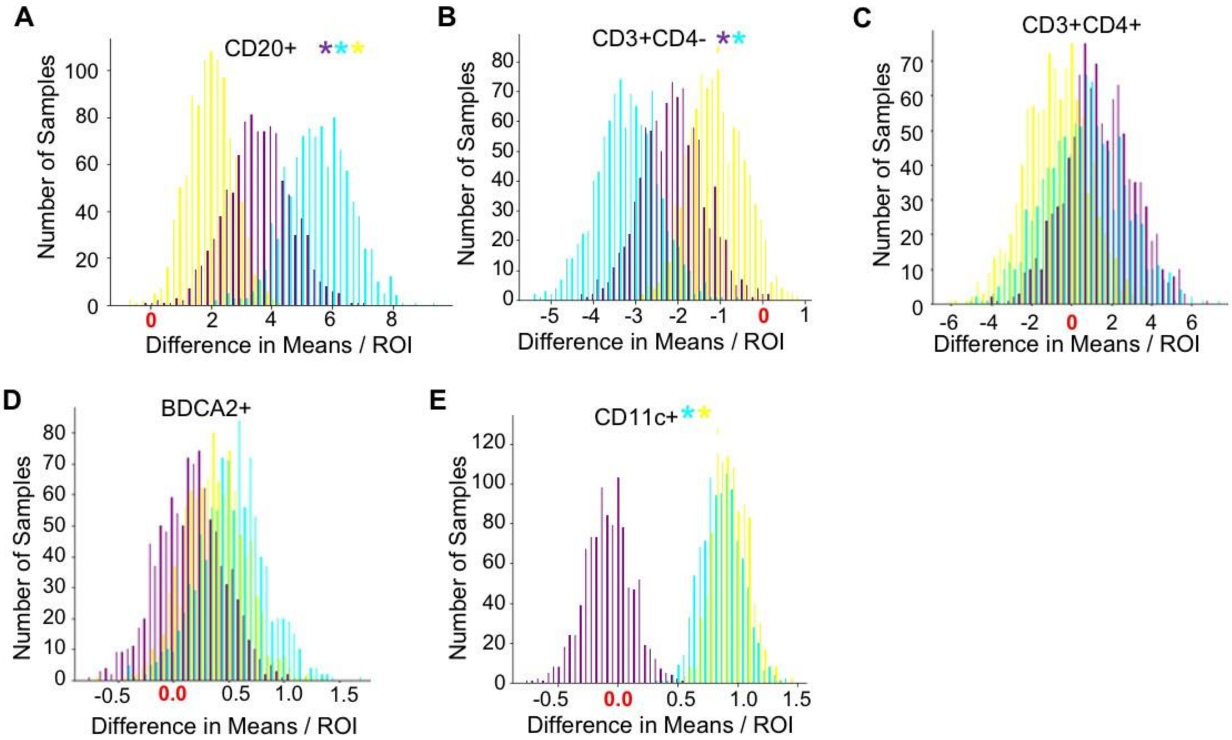

Supplementary Figure 3. Distribution of the difference in means for indicated cells. Difference in means for each bootstrapping iteration comparing ESRD+ vs ESRD- (purple), ESRD+ vs ESRD current (cyan), and ESRD- vs current (yellow) for A) CD20+, B) CD3+CD4-, C) CD3+CD4+. D) CD11c+, E) BDCA2+ cells. Stars denote the confidence intervals that do not overlap with zero, colors correspond with the comparison.

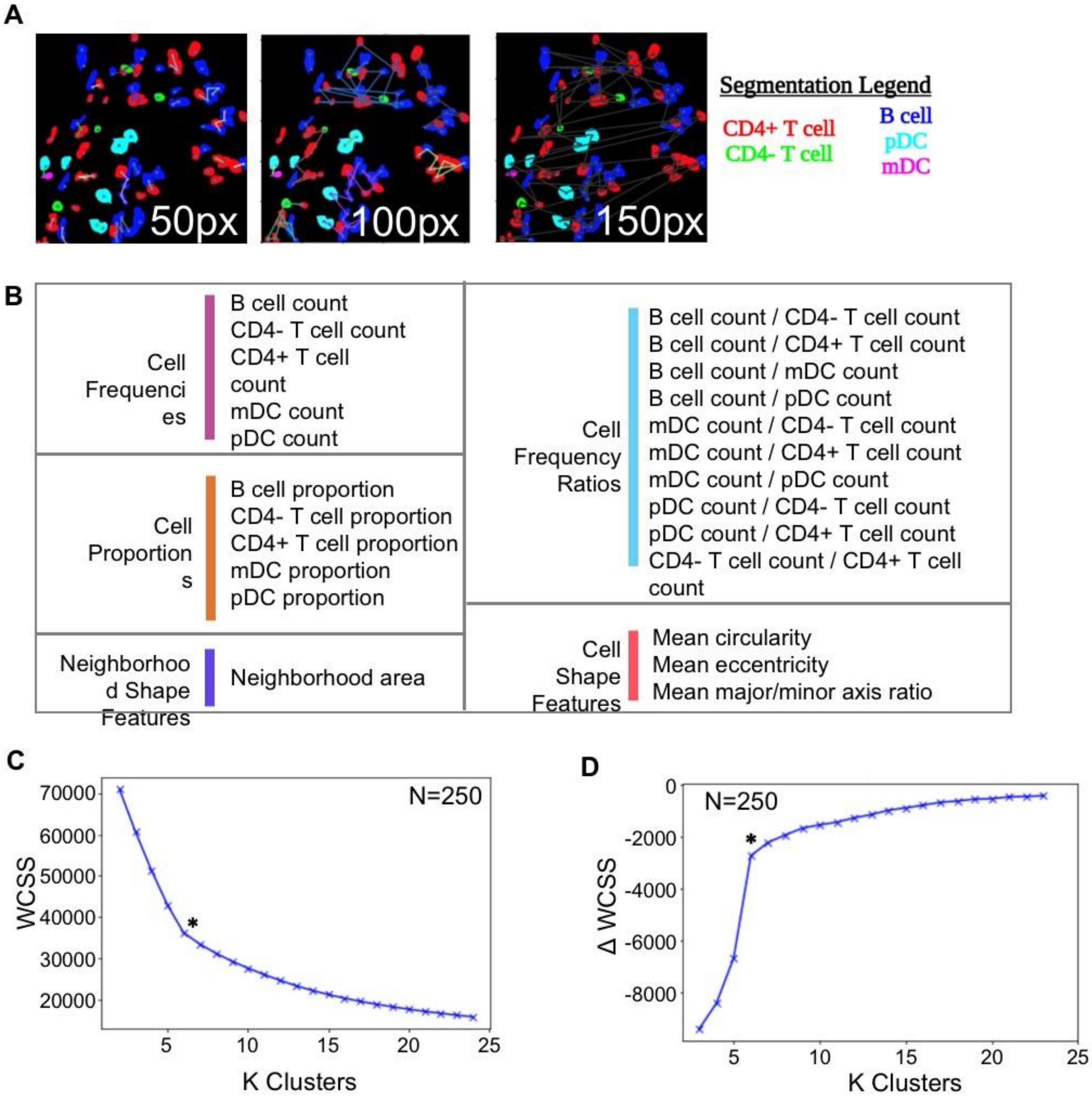
A
50px
100px
150px
Segmentation Legend
CD4+ T cell
CD4- T cell
B cell
pDC
mDC
B
Cell Frequencies
B cell count
CD4- T cell count
CD4+ T cell count
mDC count
pDC count
Cell Proportions
B cell proportion
CD4- T cell proportion
CD4+ T cell proportion
mDC proportion
pDC proportion
Neighborhood Shape Features
Neighborhood area
Cell Frequency Ratios
B cell count / CD4- T cell count
B cell count / CD4+ T cell count
B cell count / mDC count
B cell count / pDC count
mDC count / CD4- T cell count
mDC count / CD4+ T cell count
mDC count / pDC count
pDC count / CD4- T cell count
pDC count / CD4+ T cell count
CD4- T cell count / CD4+ T cell count
Cell Shape Features
Mean circularity
Mean eccentricity
Mean major/minor axis ratio
C
N=250
WCSS
K Clusters
D
N=250
Δ WCSS
K Clusters

Supplementary Figure 4. Definition of cell neighborhoods in the HR dataset. A) Representative outputs of DBSCAN algorithm with varying distance cutoffs (50, 100, and 150 pixels), B) 24 features used to define types of aggregates, results of bootstrapping method for determination of optimal cluster number using C) within cluster sum of distances squared (WCSS) and D) delta WCSS. The optimal cluster hyperparameter used for downstream analyses (k = 6), is denoted by an asterisk.

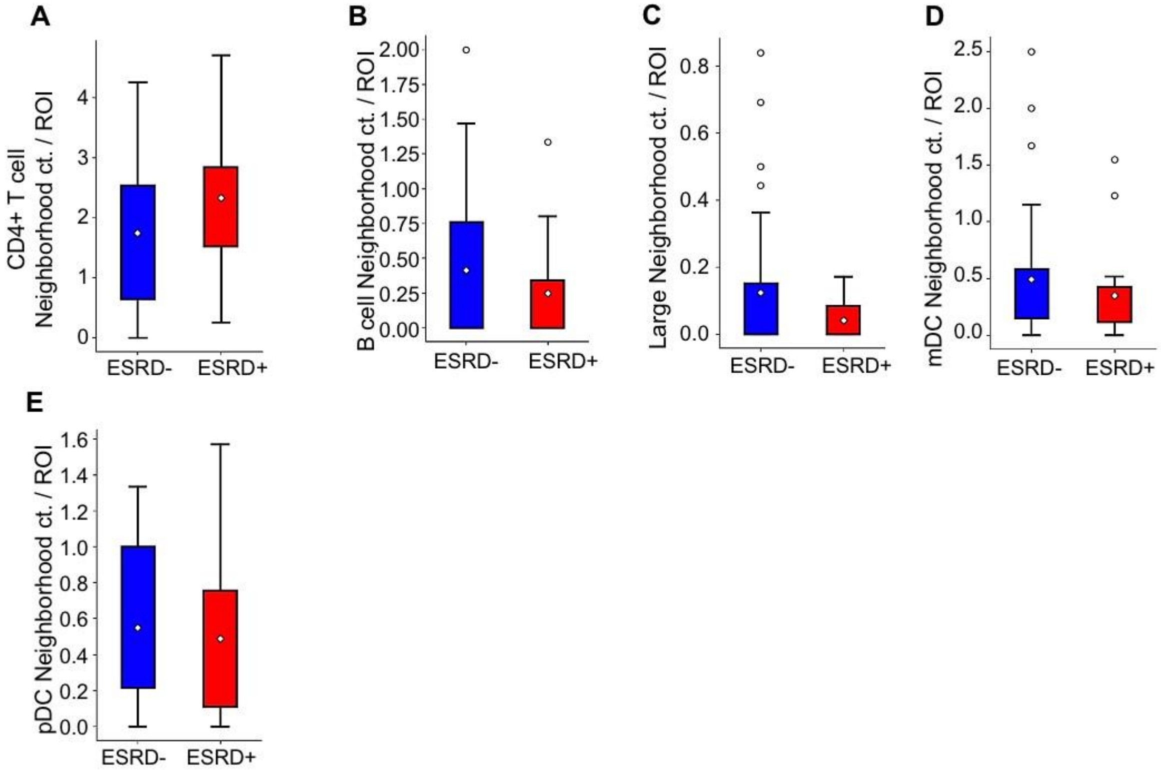

Supplementary Figure 5. Other cellular neighborhoods not associated with renal failure. The abundance of neighborhoods between the patient cohorts, normalized by the number of ROIs per patient, is compared by Mann-Whitney U Test, with a Bonferroni correction for A) CD4+ T cell neighborhoods, B) B cell neighborhoods, C) Large neighborhoods, D) mDC neighborhoods, E) pDC neighborhoods

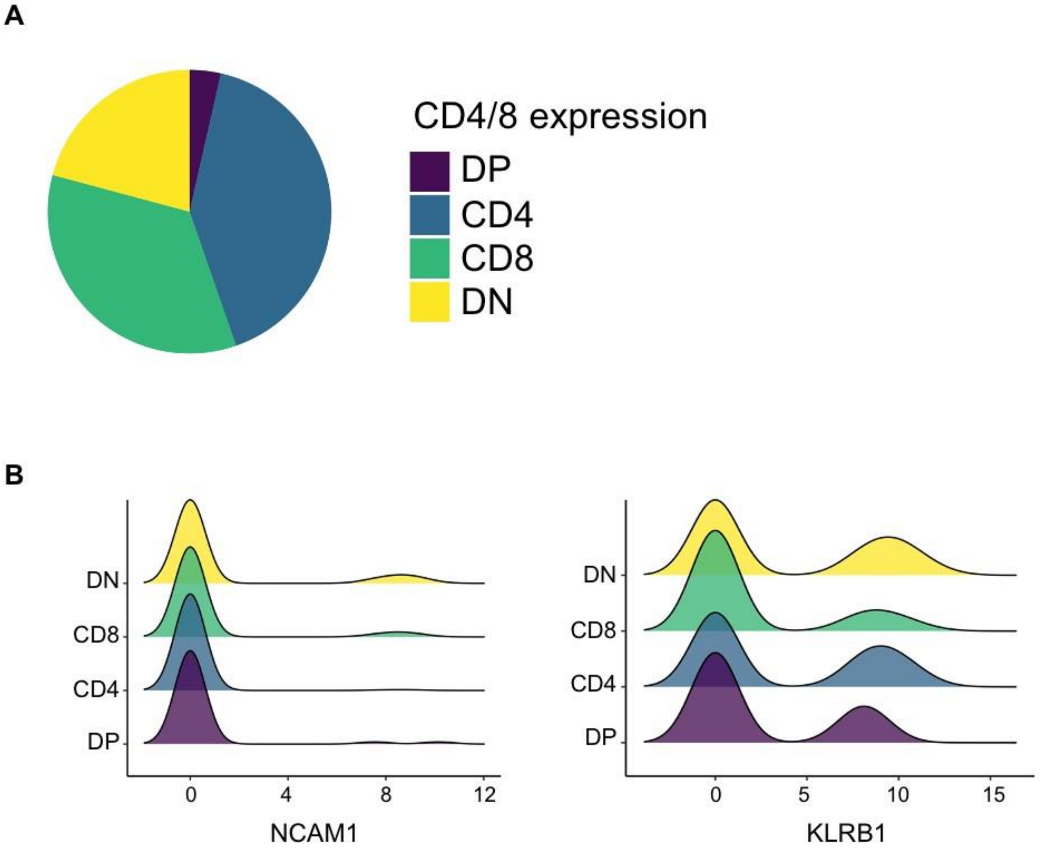

Supplementary Figure 6. DN T cells are found in scRNA-Seq LN data. A) Distribution of *CD4/8A/8B* expression in T cell population. DP: double-positive, DN: double-negative; B) Density plots showing distribution of *NCAM1* and *KLRB1* expression.

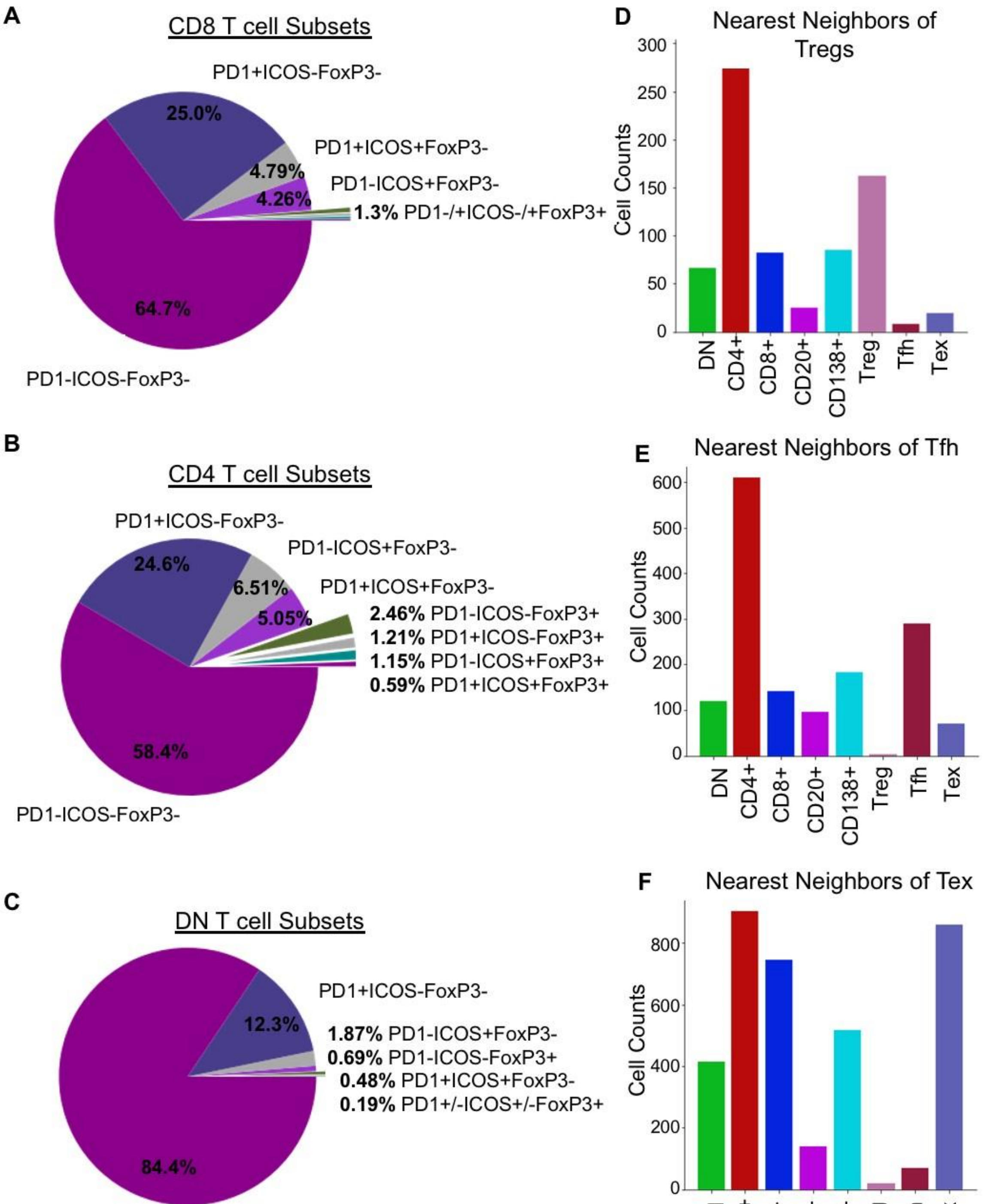
A
CD8 T cell Subsets
PD1+ICOS-FoxP3-
25.0%
PD1+ICOS+FoxP3-
4.79%
PD1-ICOS+FoxP3-
4.26%
1.3% PD1-/+ICOS-/+FoxP3+
64.7%
PD1-ICOS-FoxP3-
B
CD4 T cell Subsets
PD1+ICOS-FoxP3-
24.6%
PD1-ICOS+FoxP3-
6.51%
PD1+ICOS+FoxP3-
5.05%
2.46% PD1-ICOS-FoxP3+
1.21% PD1+ICOS-FoxP3+
1.15% PD1-ICOS+FoxP3+
0.59% PD1+ICOS+FoxP3+
58.4%
PD1-ICOS-FoxP3-
C
DN T cell Subsets
PD1+ICOS-FoxP3-
12.3%
1.87% PD1-ICOS+FoxP3-
0.69% PD1-ICOS-FoxP3+
0.48% PD1+ICOS+FoxP3-
0.19% PD1+/-ICOS+/-FoxP3+
84.4%
PD1-ICOS-FoxP3-
D
Nearest Neighbors of Tregs
Cell Counts
0
50
100
150
200
250
300
DN
CD4+
CD8+
CD20+
CD138+
Treg
Tfh
Tex
E
Nearest Neighbors of Tfh
Cell Counts
0
100
200
300
400
500
600
DN
CD4+
CD8+
CD20+
CD138+
Treg
Tfh
Tex
F
Nearest Neighbors of Tex
Cell Counts
0
200
400
600
800
DN
CD4+
CD8+
CD20+
CD138+
Treg
Tfh
Tex

Supplementary Figure 7. CD8, CD4, and DN T cell phenotypes in LN. Distribution of secondary marker (ICOS, PD1, FoxP3) expression in A) CD8+, B) CD4+ and C) DN T cells, Distribution of nearest neighbors of D) Treg (CD3+CD4+PD1-ICOS-FoxP3+) cells, E) Tfh (CD3+CD4+PD1+ICOS+/-FoxP3+) cells, and F) Tex (CD3+CD4+PD1+ICOSFoxP3-)

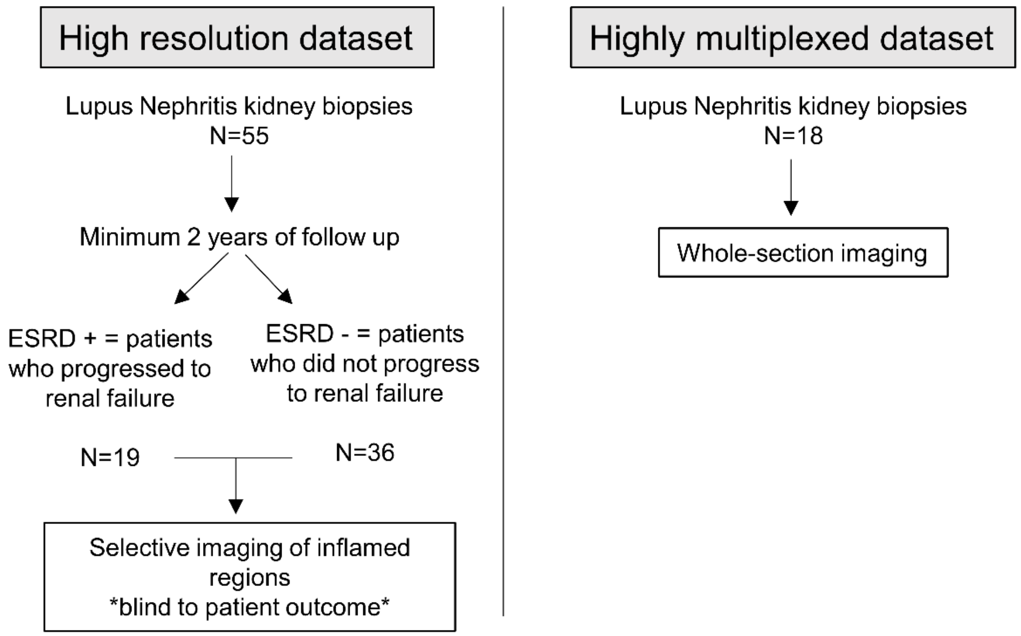

Supplementary Figure 8. Schematic of data acquisition. Left: Sample selection for the high resolution dataset. Right: Sample selection for the highly multiplexed dataset.

Supplementary Table 1 attached as an excel sheet

Supplementary Table 2. Selected clinical features from longitudinal patient cohort stratified by ESRD status.

Feature	ESRD(n=36)	ESRD+ (n=19)	p-value *(Chi-square test for independence)*
Sex			0.822
Female	33 (92%)	17 (89%)	
Male	3 (8%)	2 (11%)	
Race			0.103
African American	29 (81%)	19 (100%)	
Non-African American	7 (19%)	0 (0%)	
Induction			0.628
Cyclophosphamide	15 (42%)	9 (47%)	

MMF	14 (39%)	5 (26%)	
Pre-biopsy plaquenil	18 (50%)	13 (68%)	0.306
Pre-biopsy MMF	8 (22%)	9 (47%)	0.136
Pre-biopsy prednisone >20 mg	8 (22%)	5 (26%)	0.995
Pre-biopsy azathioprine	0 (0%)	2 (11%)	0.11
Ace inhibitor/ARB	28 (78%)	8 (42%)	***$6.37x10^{-4}$ ****
Class 5 glomerulonephritis	19 (53%)	8 (42%)	0.639
Proliferative glomerulonephritis	29 (81%)	16 (84%)	0.973
Moderate-Severe TI score (>1)	21 (58%)	17 (89%)	***0.0385 ****
Moderate-Severe chronicity score (>=4)	7 (19%)	14 (74%)	***$2.67x10^{-4}$ ****
Hypertension	17 (47%)	12 (63%)	0.400
dsDNA	30 (83%)	18 (95%)	0.435
Doubled serum creatinine during follow-up	9 (25%)	10 (52%)	0.0799

Supplementary Table 3. F1-scores for individual cell classes in the HR dataset for lymphocyte network (trained only for T and B cells), the DC network, and the combined predictions.

	Lymphocyte Network	DC Network	Joint Predictions
**CD4+ T cell**	0.75	--	0.78
**CD4- T cell**	0.69	--	0.69
**B cell**	0.84	--	0.88
**pDC**	--	0.69	0.79
**mDC**	--	0.52	0.48
**Overall**	***0.76***	***0.62***	***0.74***

Supplementary Table 4. Mean minimum distances of lymphocyte classes to structures in lupus nephritis kidney biopsies in the HMP dataset.

Cell type	Mean minimum distance to glomerulus (μm)	Mean minimum distance to tubule (μm)
DN T cell	294.61	17.82
CD4+ T cell	292.89	18.91

CD8+ T cell	299.08	16.58
CD20+ B cell	296.42	19.39
CD138+ plasma cell	299.32	15.18

Supplementary Table 5. P-values from a statistical comparison of cell class distances to glomeruli and tubules. All p-values represent the significance level of a Mann-Whitney U-Test. Significant values are underlined and bold.

Comparison	Glomerular Proximity	Tubular Proximity
DN vs. CD4+	0.0879	**1.27×10^{-14}**
DN vs. CD8+	0.279	**2.07×10^{-7}**
DN vs. CD20+	0.121	**1.22×10^{-6}**
DN vs. CD138+	**1.26×10^{-8}**	**1.30×10^{-68}**
CD4+ vs. CD8+	**0.0154**	**8.26×10^{-49}**
CD4+ vs. CD20+	**7.45×10^{-3}**	0.402
CD4+ vs. CD138+	**9.89×10^{-21}**	**2.01×10^{-223}**
CD8+ vs. CD20+	0.265	**9.29×10^{-19}**
CD8+ vs. CD138+	**2.03×10^{-7}**	**3.36×10^{-42}**
CD20+ vs. CD138+	**5.47×10^{-4}**	**8.80×10^{-70}**

Supplemental Table 6. Anti-CD20(Agilent, M0755), anti-CD4(Abcam, ab133616), antiCD3(BIO-RAD, MCA1477), anti-BDCA2(R&D, AF1376), and anti-CD11c (Abcam, ab52632) were applied for high-resolution images. For high-dimensional images, more antibodies were added, including anti-CD8(Abcam, ab17147), anti-ICOS(Abcam, ab105227), anti-PD1(Abcam, ab52587), anti-Foxp3(Invitrogen, 14-4776-82), antiCD138(Invitrogen, MA5-12400), anti-MX1(R&D, AF7946), and anti-TCRd(Santa Cruz, sc-100289). DAPI (Invitrogen, H3570) was used for nuclear staining.

Target	Vendor	Catalog#
CD20	Agilent	M0755
CD4	Abcam	ab133616
CD3	BIO-RAD	MCA1477
BDCA2	R&D	AF1376
CD11c	Abcam	ab52632
CD8	Abcam	ab17147

ICOS	Abcam	ab105227
PD1	Abcam	ab52587
FoxP3	Invitrogen	14-4776-82
CD138	Invitrogen	MA5-12400
MX1	R&D	AF7946
TCRd	Santa Cruz	sc-100289
DAPI	Invitrogen	H3570
